# Equitable colorectal cancer screening implementation framework via a modified Delphi Consensus for Romanian healthcare

**DOI:** 10.1038/s41598-025-26713-7

**Published:** 2025-11-28

**Authors:** Ionut Negoi, The SCREECO study group, Ionut Negoi, Alexandra Boromiz, Suman Baral, Eduard-Alexandru Bonci, Mihai Botezatu, Teodor Cabel, George Ovidiu Cirstea, Gabriel Constantinescu, Ionut Bogdan Diaconescu, Corneliu Dimitriu, Gabriel Dimofte, Constantin Dina, Florin Dinu, Horia Doran, Sergey Efetov, Biniam Ewnte, Ildar Fakhradiyev, Matteo Frasson, Roxana Ganescu, Dragos Garofil, Zoe Garoufalia, Octav Ginghina, Florin Grama, Claudiu Herteliu, Ioan Tănase, Victor Stefan Ionescu, Hizbullah Jan, Kenneth YY Kok, Sorinel Lunca, Robert Gabriel Lupu, Yasuko Maeda, Iuliana Marin, Radu Mihail Mirica, Alin Moldoveanu, Anca Morar, Francesk Mulita, Ana-Maria Musina, Velenciuc Natalia, Ruxandra Negoi, Omar Rabah Obaid, Akmalbek Otabekov, Francesco Pata, Tapan Patel, Gianluca Pellino, Sorin Stefan Popescu, Xenia Pucheanu, Lupu Robert Gabriel, Luis Rodrigo, Laura Elena Roșu Munteanu, Vasile Sandru, Massimo Sartelli, Haddadi Saïd, Toni Seppälä, Charalampos Seretis, Mostafa Shalaby, Makkai Silviu, Bogdan Stoica, Kire Stojkovski, Marcel Tantau, Ciprian Toma, Elena Adelina Toma, Alexandru-Vicentiu Tudor, Stefana Ilinca Tudorascu, Alexia Ungureanu, Andreea-Catalina Ungureanu, Gabrielle van Ramshorst, Georgios Ioannis Verras, Albina Zubayraeva

**Affiliations:** 1 Carol Davila University of Medicine and Pharmacy Bucharest, Clinical Emergency Hospital of Bucharest, No 8 Floreasca Street, Sector 1, 014461 Bucharest, Romania; 2 Carol Davila University of Medicine and Pharmacy Bucharest, Bucharest, Romania; 3 Dirghayu Pokhara Hospital, Pokhara, Nepal; 4 Iuliu Hatieganu University of Medicine and Pharmacy, Cluj-Napoca, Romania; 5 Clinical Emergency Hospital of Bucharest, Bucharest, Romania; 6 Alexandria County Emergency Hospital, Alexandria, Romania; 7 Jersey General Hospital, Jersey, United Kingdom; 8 Regional Institute of Oncology, University of Medicine and Phramacy Grigore T. Popa, Iasi, Romania; 9 Ovidius University, Constanta, Romania; 10 Public Involvement, Bucharest, Romania; 11 IM Sechenov First Moscow State Medical University, Moscow, Russia; 12 Debre Tabor University, Debre Tabor, Ethiopia; 13 Kazak National Medical University, Almaty, Kazakhstan; 14 University Hospital La Fe, Valencia, Spain; 15 KKH Prignitz, Perleberg, Germany; 16 Cleveland Clinic Florida, Cleveland, Greece; 17 Saint John Emergency Clinical Hospital, Bucharest, Romania; 18 Coltea Clinical Hospital, Bucharest, Romania; 19 Bucharest University of Economic Studies, Bucharest, Romania; 20 Khyber Medical College, Khyber, Pakistan; 21 Pengiran Anak Puteri Rashidah Saadatul Bolkiah Institute of Health Sciences, Universiti Brunei Darussalam, Darussalam, Brunei; 22 Regional Institute of Oncology Iasi, Iasi, Romania; 23 Gheorghe Asachi Technical University of Iasi, Iasi, Romania; 24 Queen Elizabeth University Hospital, Glasgow, United Kingdom; 25 University Politehnica of Bucharest, Bucharest, Romania; 26 Saint John Emergency Hospital Bucharest, Bucharest, Romania; 27 Department of Surgery, General University Hospital of Patras, Patras, Greece; 28 University of Medicine and Pharmacy Grigore T. Popa/Regional Institute of Oncology Iasi, Iasi, Romania; 29 Department of Surgery, Nicola Giannettasio Hospital, Corigliano-Rossano, Corigliano-Rossano, Italy; 30 Baroda Medical College and SSG Hospital, Baroda, India; 31 Department of Advanced Medical and Surgical Sciences, Università degli Studi della Campania Luigi Vanvitelli, Naples, Italy; 32 Zarnesti Hospital, Zarnesti, Romania; 33 HUCA, Oviedo, Spain; 34 Clinical Emergency Hospital of Bucharest, Bucharest, Romania; 35 Macerata Hospital, Macerata, Italy; 36 Central Hospital of the Army, Lucknow, Algeria; 37 University of Tampere/Helsinki, Tampere, Finland; 38 St Andreas General Hospital of Patras, Patras, Greece; 39 Mansoura University, Mansoura, Egypt; 40 Regina Maria Brasov, Brasov, Romania; 41 PSI-CRO, Skopje, Macedonia; 42 University of Medicine and Pharmacy Cluj-Napoca, Cluj-Napoca, Romania; 43 Ghent University Hospital, Ghent, Belgium; 44 General University Hospital of Patras, Patras, Greece

**Keywords:** Colorectal cancer, Screening, Radical resection, Healthcare disparities, Delphi consensus, Guidance, Screening adherence, Early detection, Disease prevention, Health policy, Health services, Public health, Quality of life, Cancer, Colonoscopy, Gastrointestinal system

## Abstract

Colorectal cancer (CRC) continues to be a predominant cause of cancer-related mortality on a global scale. Screening for early detection plays a crucial role in significantly reducing both incidence and mortality rates. However, screening uptake varies widely across regions due to disparities in healthcare resources and implementation of screening programs. The aim of this study was to evaluate various potential strategies for implementing an effective colorectal cancer screening program that enhances adherence. This program should be feasible and appropriate, considering the demographic characteristics, geographical distribution, and logistical factors, including human and material resources, of medical facilities at the national level in Romania. A multidisciplinary panel was convened using a modified Delphi process (two survey rounds and a consensus meeting in early 2022) to develop a harmonized CRC screening framework tailored to diverse healthcare settings. Clinicians and allied professionals from 19 countries (Europe, Asia, and Africa) contributed to the consensus recommendations for average-risk individuals. The panel advised that primary care physicians should play a central role in CRC risk assessment and patient engagement and endorsed initiating screening at age 45 and continuing to 75, with selective extension up to 85 for healthy, willing individuals. To improve participation, we recommended region-specific screening strategies that accommodate local resources and demographics and emphasized a patient-centered approach offering multiple modalities (colonoscopy, fecal immunochemical testing, CT colonography), emphasizing that the most effective test is one that patients are willing to complete. Finally, the study group deemed implementing a centralized system for screening invitations, result tracking, and patient recall essential. This consensus guideline provides a scalable model to enhance screening adherence and reduce CRC mortality through early detection and prevention. This framework is developed for the Romanian healthcare context, informed by international professional input but tailored to local healthcare infrastructure and estimated population needs.

**Supplementary Information:**

The online version contains supplementary material available at 10.1038/s41598-025-26713-7.

## Introduction

Colorectal cancer (CRC) remains a leading global malignancy, accounting for approximately 1.9 million new diagnoses in 2020, representing 9.8% of all cancer cases. It is also a major contributor to cancer-related mortality, with approximately 935,000 deaths globally, representing 9.4% of all cancer fatalities^1^. Although it predominantly affects older adults, with the majority of cases occurring after the age of 50 years, recent evidence has shown that the incidence in 40–49 years cohorts increased by 15% during the last two decades^2,3^.

CRC is largely preventable and curable if detected at an early stage. Most tumors develop from adenomatous polyps over a period of 10–20 years, providing a critical window for intervention^4^. Screening can identify and remove precancerous polyps or detect early-stage cancer, thereby significantly reducing CRC incidence and mortality^5^. Indeed, long-term studies and meta-analyses have demonstrated that implementing CRC screening programs leads to a marked decline in CRC cases and deaths in screened populations^5^. Approximately 40% of CRC cases are currently diagnosed at a localized, early stage, and improving screening uptake is vital to increase early detection rates^4^. In recognition of these benefits, numerous international guidelines have been established to promote CRC screening in average-risk populations. Most guidelines worldwide recommend initiating screening by age 50 and continuing until approximately 75 years, using either high-sensitivity stool tests or structural investigations of the colon^6^.

For asymptomatic adults at average risk, standard colorectal cancer screening protocols generally include fecal occult blood testing (FOBT), which may be either guaiac-based or immunochemical (FIT), conducted on an annual or biennial basis. Additionally, endoscopic methods, such as flexible sigmoidoscopy every five years or colonoscopy every ten years, are widely endorsed options^6^. In recent updates, some organizations have lowered the starting age to 45 years in response to the rising early onset CRC incidence. For instance, the American Cancer Society now recommends screening for individuals aged 45 to 75, with personalized decisions extending up to age 85 based on their health status^7^. Similarly, the U.S. Preventive Services Task Force identifies sufficient benefits to initiate screening at age 45, while reaffirming the strong recommendation for individuals aged 50–75^2^.

Despite the proven efficacy of CRC screening, there remain significant disparities in its implementation and uptake across different regions and populations, with reported participation rates ranging from 16% to 68.2%^8^. Even in the United States, with longstanding screening recommendations, 26% of eligible adults have never been screened and 31% are not up-to-date with recommended screening, indicating substantial gaps in adherence^2^. Socioeconomic and racial/ethnic disparities further contribute to inequitable screening uptake, with individuals from lower socioeconomic status or minority communities consistently demonstrating lower CRC screening rates and higher late-stage diagnoses, both in high-income and low-income countries^9^.

Globally, factors such as resource availability, healthcare infrastructure, public awareness, and cultural attitudes towards screening influence the success of CRC screening programs. It is increasingly recognized that one-size-fits-all approaches may not achieve optimal coverage, and guidelines emphasize tailoring screening strategies to local contexts and patient preferences to improve participation, considering that any screening is considered better than none and that ”trust” and ”building trusted relationships” are factors that contribute significantly to a successful screening program^10^.

The aim of the current study was to analyze diverse possible approaches for an effective and adherence-enhancing colorectal cancer screening program that may be feasible and suitable for the demographic characteristics, topography, location, and logistical considerations, including human and material resources, of medical facilities at the national level in Romania. Considering the global challenges in CRC screening uptake, our study focuses on Romania to explore context-specific strategies. Romania, like several Central European countries, faces a rising CRC burden, but has limited screening coverage to date. We aimed to develop a framework for CRC screening that aligns with the country’s demographics, healthcare resources, and logistical realities while maximizing adherence in the target population. By leveraging professional consensus and evidence-based practices, this study sought to design a feasible, high-yield screening program that can ultimately reduce CRC mortality in Romania and serve as a model for similar settings.

Romania faces unique challenges in colorectal cancer control, with incidence and mortality rates among the highest in the European Union^11^. Despite European recommendations for population-based screening programs, Romania has implemented only pilot CRC screening initiatives (ROCCAS project) in select regions between 2018-2019, with no comprehensive national program established^12^. The Romanian healthcare system is characterized by fragmented primary care services, significant disparities between urban and rural healthcare access, and limited public health infrastructure for organized screening programs. Romania was up to 2024 one of only two EU Member States without a national colorectal cancer screening program^13^, highlighting the urgent need for a tailored implementation framework. This study addresses this gap by developing consensus recommendations specifically adapted to Romanian healthcare realities while incorporating international best practices.

This study does not encompass the entirety of available evidence or all screening modalities for colorectal cancer, nor does it account for every clinical nuance, comorbidity, or individualized patient presentation. Its application should be tailored to align patient-specific preferences and clinical judgments. The proposed framework is intended as an optional approach for clinical implementation and must be integrated with independent professional evaluations. While the present work is consistent with the prevailing medical literature, it acknowledges the inherent variability within existing data and should be considered a guiding reference rather than an absolute standard of care. Neither the author nor the study group endorse any specific external services, therapeutic interventions, medical devices, or pharmacologic agents. Furthermore, they disclaim any responsibility for potential adverse outcomes arising from the use of this information^14^.

## Results

**Figure 1:**
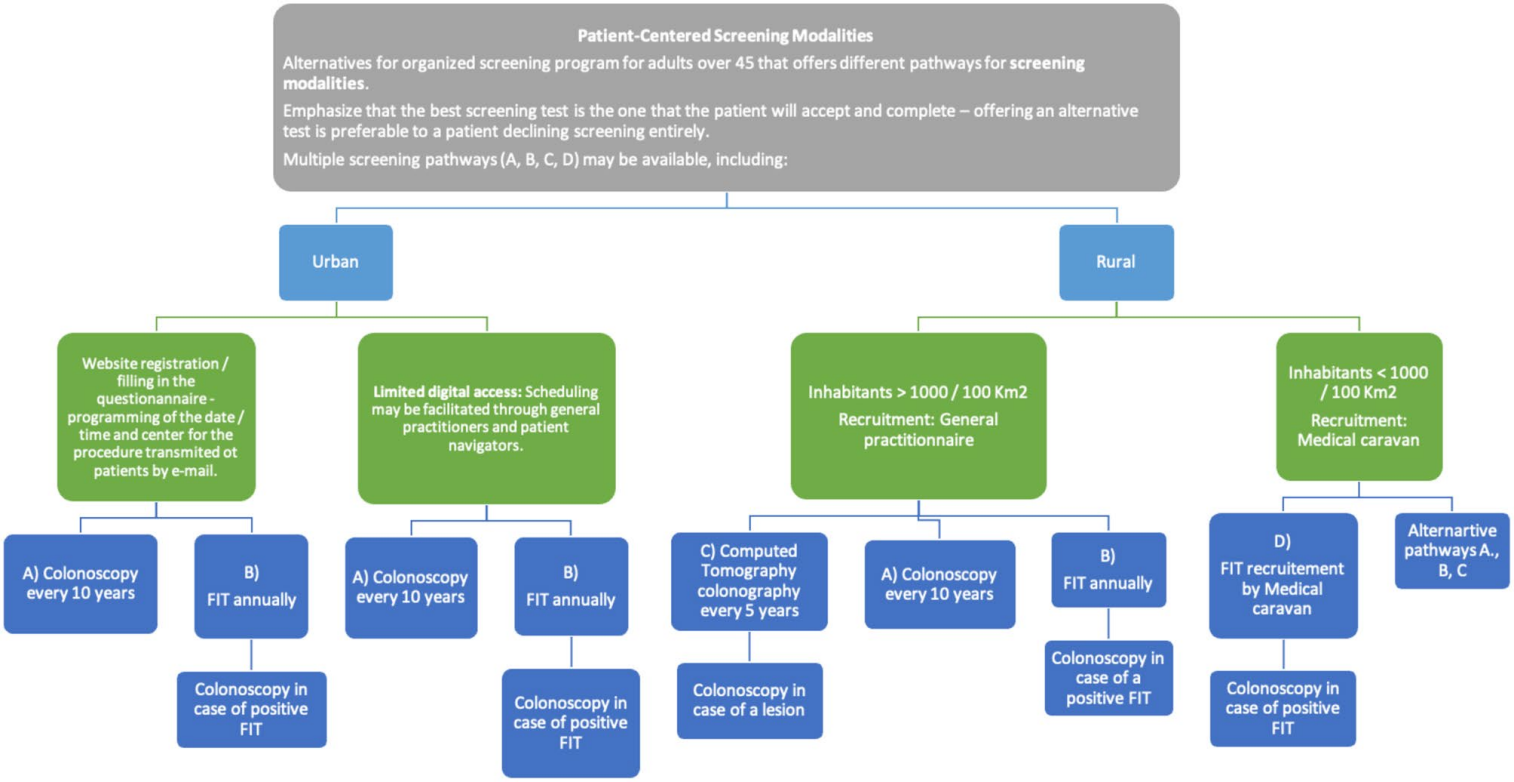
Proposed algorithm for colorectal cancer screening.

The final statements after the two voting rounds and in-person conference are summarized in Table 1, and the proposed screening algorithm is illustrated in Figure 1.

**Table 1:** Final statements after the two voting rounds and the in-person conference in Equitable Colorectal Cancer Screening Implementation Framework via a Modified Delphi Consensus for Romanian Healthcare – SCREECO project.

**Statement 1** Primary Care Risk Stratification: Primary care physicians may assume a central role in the assessment of colorectal cancer (CRC) risk, initiating risk stratification at approximately 35 years of age and repeating it every 3 years thereafter.
**Statement 2** Standard Risk Assessment Factors: Routine risk assessment in primary care may include evaluation of the subsequent factors: • Familial or personal history of CRC or adenomatous polyps. • Family history of hereditary syndromes associated with CRC (e.g., familial adenomatous polyposis, Lynch syndrome). • Personal history of inflammatory bowel disease, specifically ulcerative colitis or Crohn’s disease. • Personal history of abdominal radiation for a pediatric cancer. • Patients with none of these risk factors are classified as average-risk and may undergo standard screening, whereas those with any of these factors may follow high-risk screening protocols.
**Statement 3** Screening Initiation and Age Limits: For individuals at average risk, CRC screening may start at the age of 45 and continue until the age of 75.. In individuals who remain healthy and fit, it is reasonable to extend screening beyond 75 years up to 85 years on a case-by-case basis, considering overall health and patient preference.
**Statement 4** Tailored Regional Strategies: Implement region-specific screening strategies to maximize uptake. Programs may be tailored to local resources and population needs (for example, distinct approaches in urban vs. rural communities) to ensure broad participation in screening.
**Statement 5** Patient-Centered Screening Modalities: alternatives for organized screening program for adults over 45 that offers different pathways for screening modalities. It is crucial to highlight that the most effective screening test is one that the patient is willing to accept and complete. Offering an alternative test is preferable to the patient declining screening altogether. Multiple screening pathways may be available, including: **Urban areas:** i. Digital access: Patients may register online, complete a screening questionnaire, and receive scheduling details via email. • Pathway A: Colonoscopy every 10 years. • Pathway B: Annual fecal immunochemical test (FIT), with colonoscopy for positive results. ii. Limited digital access: Scheduling may be facilitated through general practitioners and patient navigators. • Pathway A: Colonoscopy every 10 years. • Pathway B: Annual FIT, with colonoscopy for positive results. **Rural areas:** i. Regions with >1000 inhabitants per 100 km^2^: Recruitment may be managed by general practitioners. • Pathway A: Colonoscopy every 10 years. • Pathway B: Annual FIT, with colonoscopy for positive results. • Pathway C: Computed tomography colonography every 5 years, with colonoscopy for detected lesions. ii. Regions with <1000 inhabitants per 100 km^2^: Mobile medical units may facilitate screening. • Pathway D: FIT-based screening, with colonoscopy for positive results. • Alternative pathways (A, B, or C) may be utilized as feasible.
**Statement 6** Continuation of a National Organized Program for coordinated CRC screening program with standardized guidelines and oversight: is beneficial for a centralized program ensuring quality control, consistency in screening practices, and equitable access across different regions.
**Statement 7** Centralized Tracking and Recall System: A centralized information system to support the screening program is needed. This system may automate the sending of invitations, track test results, flag and recall patients who are overdue for screening, and direct patients to appropriate screening centers or referral hospitals. Such infrastructure is essential to improve screening participation rates and to ensure timely follow-up of abnormal findings.

### Participant characteristics and survey response

A total of 66 respondents participated in Delphi Round 1 (SCREECO I) (see Appendix A, Table A. 1 and Appendix C.1) and 34 in Round 2 (SCREECO II) (see Appendix A, Table A.2 and Appendix C.2). Most Round 2 participants were a subset who also responded to Round 1.

**Figure 2:**
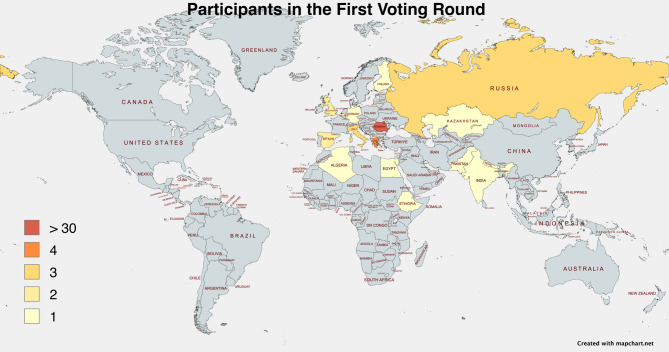
World map of participants in the first survey round.

The participants represented 19 countries across Europe, Asia, and Africa (see Figure 2). The largest contingent was from Romania (n = 40, 60.6% of round 1), reflecting the national focus of the project, followed by contributions from Italy (n = 3), Greece (n = 4), Russia (n = 3), Spain (n = 2), the UK (n=2), and one participant from each country, including Germany, Belgium, Finland, Egypt, Algeria, India, Nepal, Pakistan, Brunei, Ethiopia, Macedonia, and Kazakhstan.

Respondents encompassed a broad range of backgrounds, including general surgery, gastroenterology, imagistics, medical students, the general population, and others (62.1%, 7.6%, 1.5%, 12.1%, 3.0%, and 13.6%, respectively). Most respondents (75.8%) worked in a university/academic hospital and had a mean age of 38.4+/−11.5 years.

**Figure 3:**
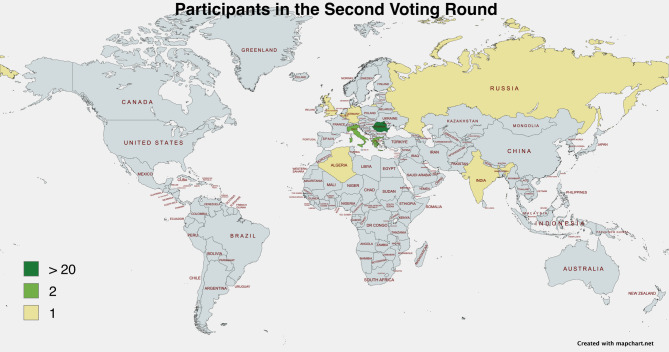
World map of participants in the second survey round.

Round 2 (SCREECO II) focused on contentious or unclear points, presenting seven key statements for consensus decisions, all phrased as recommendations. The response rate was lower (34 participants) but still with multidisciplinary and international representation (82% from Romania) (see Figure 3).

### Current CRC screening landscape

Of the 66 respondents, 23 (35.9%) reported that their country had a functional national CRC screening program. An additional eight participants (12.5%) indicated that there was a regional or pilot program, but not a nationwide program. The remaining 33 (51.6%) participants stated that no organized screening program was in place in their country.

When those with a program were asked to rate their effectiveness on a scale of 1 (poor) to 10 (excellent), the responses were modest; the median rating was 6.0, with an average of 5.7.

### Delphi results

#### Regarding the role of general practitioners (GPs) in risk assessment

97.0% agreed that primary care physicians should play a central role in CRC risk assessment, assessing patients’ risks at their visit to the GP. The first risk stratification should be performed at the age of 35 (60.6% of responders) and then reevaluated every 3 years (54.5% of responders in the first round and 73.5% after the second voting).

#### Standard risk factors to assess

**Figure 4:**
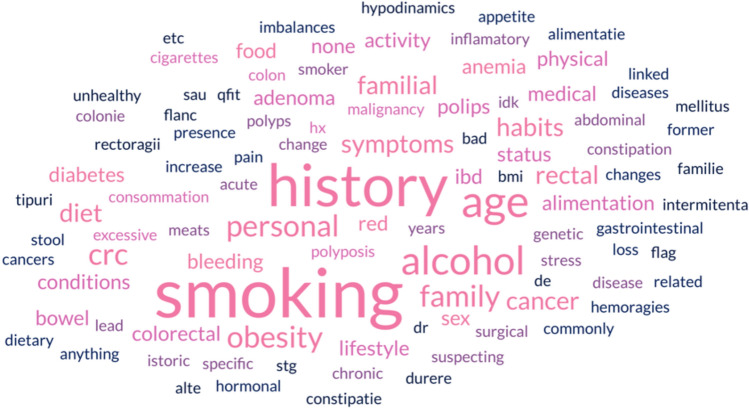
Word cloud graph with responses to question ”Q 6: Suggested risk factors to be included in the general practitioner evaluation”.

Although we observed a distribution of responses in the first round (see Figure 4), there was a strong consensus after the second round on which risk factors define a “high-risk” individual who might need earlier or more intensive screening.

97.1% agreed that a GP’s risk assessment should include the following factors: (1) familial history of cancer or polyps; (2) individual history of CRC or adenomatous polyps; (3) familial history of genetic syndromes associated with CRC; (4) personal history of ulcerative colitis or Crohn’s disease; and (5) previous abdominal radiotherapy for pediatric cancers. Patients without any of these risk factors should be classified as average-risk and undergo “standard” screening (75.8% of responders), as opposed to specialized high-risk protocols.

#### Screening start and stop ages for average risk

Round 1 responses indicated broad support for starting average-risk screening earlier than the age of 50. After the second round of voting, there was a consensus of 88.3% for starting screening in average individuals aged 45 years. There was more uncertainty about when to discontinue screening: after the second round, 61.8% agreed with stopping at 75, and some advocated a higher cutoff, especially if health status was good.

#### Preferred screening methods

Participants were asked in Round 1 (Q12), in which screening modalities should be considered for national implementation (multiple selections allowed). FIT was the most frequently endorsed method (80.3%) followed by colonoscopy (67%). Approximately 33.3% selected gFOBT, 18.1% selected flexible sigmoidoscopy, and 22.7% selected CT colonography. Many participants selected more than one modality, reflecting that a program may use a combination of modalities (e.g., a stool test for primary screening and colonoscopy for follow-up). The high endorsement of FIT and colonoscopy aligns with current evidence, FIT for its ease and cost-effectiveness, and colonoscopy for its diagnostic and preventive power. Fewer endorsed older methods, such as gFOBT, or resource-intensive approaches, such as CT colonography. These preferences led to consensus discussions regarding the screening algorithm.

#### Perceived test performance

Round 1 included questions asking participants to estimate the sensitivity of various screening tests for detecting CRC (Q13) and the number of deaths prevented per 1000 people screened with each method (Q14), based on published data. The responses revealed variability in perceptions. For instance, for annual FIT sensitivity, the most common estimate chosen was “75–80%” (by 37.7% of respondents), followed by “70–75%” (27.9%); smaller groups chose higher ranges. For colonoscopy sensitivity, the answers were balanced for every interval of over 70%. For Q14 (deaths prevented per 1000 screened), the answers were also balanced for different answers.

#### Personal and public screening strategy preferences

We asked several questions about preferred screening strategies in different scenarios (Q15–Q18). When asked, “*what screening strategy would you choose for yourself as a patient*” (average-risk scenario, Q15), the most popular choice (50.0% of respondents) was a noninvasive test-first strategy: annual FIT or periodic CT colonography with colonoscopy only if the initial test was positive. A substantial proportion (around 36.4%) chose direct colonoscopy every 10 years approach for themselves, and 13.6% chose other strategies (such as sigmoidoscopy-based options or capsule colonoscopy). This result confirms that even among many specialists, less-invasive screening was preferable, likely due to convenience and safety considerations, whereas a significant percentage (especially some surgeons and gastroenterologists) trusted colonoscopy for maximal assurance. We observed a trend by specialty; surgeons were more likely to opt for colonoscopy (approximately 44% of surgeons chose colonoscopy vs. 34% FIT-based), whereas nearly all medical students and patient representatives opted for noninvasive strategies.

To maximize patient adherence in the urban population (Q16), 54.5% of the participants recommended an FIT/CT-based strategy over colonoscopy. In rural populations (Q17), this was even higher (over 60% favored stool-based strategies), likely because rural areas often have less access to endoscopy services. Only 25.7% of the participants believed that colonoscopy-centric screening would achieve maximum adherence in rural communities. These opinions highlight the importance of accessibility: simpler methods are thought to yield better participation, especially when medical facilities are sparse. In the case of limited access to colonoscopy (Q18), which is applicable to many low-resource settings, 53% would rely on stool tests (FIT or even older guaiac tests), reserving colonoscopy for those who test positive or have symptoms. Approximately 18.2% of patients would use CT colonography every 5 years if colonoscopies are scarce. This indicates a consensus that some screening is far better than none. If colonoscopy capacity is a bottleneck, a program should lean on fecal tests to cover the population.

#### Resource and system questions

Several items probed health system considerations. When asked about the cost of colonoscopy (Q20), the responses varied; many provided estimates in the range of 100–400 euros. On whether public insurance reimbursement currently covers the true cost of colonoscopy (Q21), 25.8% answered “No”, indicating that in many places the funding for colonoscopy screening (considering costs of prep, sedation, pathology, etc.) is insufficient. Nearly all respondents (93.9%) agreed that fully reimbursing screening colonoscopies and associated early treatments would ultimately be cost-saving for the healthcare system (Q22) by reducing the costs of treating advanced cancers and preserving productivity. Regarding the strategy of starting screening directly with primary colonoscopy in high-risk individuals (as defined by general practitioners, Q23), opinions accounted for 84.8%. A consensus of 87.9% was that all available healthcare providers and facilities should be involved in delivering the screening program (Q24), with a multicenter approach rather than a single specialized center, due to the volume of the population to cover. For the question of how many people should be covered by one “screening unit” (Q25), the responses ranged, but the mean +/- standard deviation was 25162+/−41875, and the median was 6000 people per screening center. Using Delphi feedback, we estimated that each dedicated screening center (with a team and colonoscopy suite) might serve a 30–50,000 population to achieve regular screening. Finally, most respondents agreed that it is appropriate and necessary to implement a national IT system for screening invitations and scheduling (Q26), learning from countries with organized programs that use registries and recall systems. Many have pointed to the need for centralized software to send invitations, track results, and recall patients, which would greatly improve participation and follow-up.

#### Screening strategies and pathways

A composite statement was introduced in round 2, proposing distinct screening pathways tailored to urban versus rural populations (considering population awareness levels). This finding was based on Round 1 suggestion that a single size may not fit all. For urban areas, a more colonoscopy-centered approach could be considered, whereas for rural populations, a noninvasive approach might yield better adherence. This statement essentially endorses the dual-strategy algorithm. This received 85.3% agreement in round 2, indicating a consensus (see Figure 1).

## Discussion

In the current study, we proposed a tailored approach for CRC screening strategies in different areas of the country, correlated with population demographics, disease burden, and regional healthcare system organization.

### Economic considerations and screening intervals

The panel’s initial recommendation for annual FIT screening reflects evidence showing superior cost-effectiveness under conditions of high adherence. Recent systematic analyses have indicated that annual FIT screening results in the most significant increase in quality-adjusted life years and is more economically advantageous compared to the absence of screening^15,16^. However, practical implementation considerations in healthcare systems with limited resources favor biennial screening intervals, as adopted by most European programs^17^.

Romanian screening strategy published in 2024 by the Romanian Ministry of Health considers biennial FIT as the primary strategy^13^. However, assuming realistic levels of adherence, conducting annual FIT proved to be the most effective and economical approach^16^. Cost-effectiveness modeling specific to Romanian healthcare costs and epidemiology will be essential for optimizing the screening interval. Studies from similar healthcare systems suggest biennial FIT remains highly cost-effective, with incremental cost-effectiveness ratios well below accepted thresholds^15,17,18^. The framework allows flexibility in screening intervals based on regional capacity and performance monitoring.

### Comparison with international models

Internationally, there are two models for CRC screening: organized and opportunistic (see supplementary material Table A.3). Organized screening is a dedicated program for CRC screening in which the population is invited for screening, in mass, at a specific age, and follow-up in the long term. Opportunistic screening is used to investigate when patients are admitted for other reasons^19^. Globally, the most common method for CRC screening is FIT, whereas in the USA and several other regions, including parts of Germany and Poland, colonoscopy is the preferred screening technique^19^.

Our consensus guidelines emphasize the role of primary care in CRC screening, aligning with findings from both developed and developing healthcare systems that an international Comparison of Programs revealed that 54% of our participants reported no national screening in their country, highlighting the global gap. Regions represented by our respondents with no programs included parts of Eastern Europe, Central Asia, and North Africa. Those from countries with established programs (such as Italy, the UK, Finland, and Belgium) generally rated them as moderately effective. This aligns with reports that even in the EU, many programs have not yet reached high coverage. For instance, one participant from Spain commented that their program was “functional but could be improved: participation approximately 50%”. Participants from countries such as Nepal, Ethiopia, and Algeria pointed out that they rely on opportunistic screening, and that there is much room for improvement.

Analysis of the European Union Member States revealed a wide variability in screening performance, with a participation rate higher for fecal immunochemical testing (FIT) (interval 22.8% - 71.3%) than for guaiac fecal occult blood testing (gFOBT) (4.5 - 67%) (P < 0.001)^20^. Compliance with colonoscopy varies between 64% and 92%, with a completion rate of 92–99%^20^.

In our study, we observed specialty differences, with certain opinions varying according to specialty. Surgeons and gastroenterologists, for example, were more confident about colonoscopy (nearly half of the surgeons chose colonoscopy for their own screening). Non-physician participants leaned more towards stool tests. This may reflect knowledge of the test characteristics and personal comfort; providers know the benefits of colonoscopy but are also aware of its burdens, whereas patients favor less invasive options. Importantly, despite these differences, the consensus process led both groups to agree on an FIT-first approach to population screening, recognizing the need to maximize participation.

High-quality studies have consistently demonstrated CRC mortality reduction through screening compared with no screening, notably using gFOBT (relative risk 0.78–0.91), FIT (relative risk 0.90), flexible sigmoidoscopy (incidence risk ratio 0.74), colonoscopy (0.32, 95% CI 0.24–0.45), and CT colonography (sensitivity 0.86–1.0)^21^.

Our analysis suggests that even among health professionals, their understanding of screening test performance is not uniform. This indicates an opportunity for education as part of implementing a program, not only for the public but also for healthcare providers, so that they can uniformly counsel patients.

The long-term analysis of the UK Flexible Sigmoidoscopy Screening randomized controlled trial showed that once-only flexible sigmoidoscopy was associated with protection for more than 17 years^22^. The reduction in CRC incidence and mortality was observed to be 26% (hazard ratio [HR] = 0.74, 95% CI 0.70–0.80, P < 0.0001) and 30% (HR = 0.70, 95% CI 0.62–0.79, P < 0.0001), respectively, in the intention-to-treat analysis. In the per-protocol analysis, the reductions were 35% (HR = 0.65, 95% CI 0.59–0.71) and 41% (HR = 0.59, 95% CI 0.49–0.70), respectively^22^.

A critical benefit of CRC screening programs is the significant shift towards early-stage disease at diagnosis, enabling improved prognosis and treatment outcomes^23^. Analysis of the Kaiser Permanente Northern California health plan revealed that screening was associated with a decrease in advanced CRCs from 45.9 to 29.3 cases/100000 (P < 0.01) and a 52.4% reduction in mortality from 30.9 to 14.7 deaths/100000 (P < 0.01)^23^.

CRC screening improves OS and DFS by detecting cancer at an early stage and preventing advanced disease^24^. An RCT investigated the effect of a single screening colonoscopy performed in healthy individuals aged 55–64 years compared with no screening (11843 versus 56365 patients)^25^. After a decade, the incidence of colorectal cancer (CRC) was 0.98% in the screening cohort compared to 1.20% in the non-screening cohort, yielding a risk ratio (RR) of 0.82 (95% CI 0.70–0.93). The overall mortality rates were comparable between the screening and non-screening groups, at 11.03% and 11.04%, respectively (RR = 0.99, 95% CI 0.96–1.04). The cancer-specific mortality rates were 0.28% for the screening group and 0.31% for the non-screening group (RR = 0.90, 95% CI 0.64–1.16). To prevent one case of CRC, it was necessary to screen 455 individuals (95% CI 270 - 1429)^25^. A modified per-protocol analysis of individuals who had a colonoscopy indicated that the likelihood of dying from CRC was reduced by half in the screening group compared to the non-screening group (0.15 versus 0.30, risk ratio – 0.50, 95% CI 0.27–0.77)^25^.

### Implications for healthcare policy

Compared to Western Europe, populations from Eastern, Southern, and Northern Europe were less likely to be updated with CRC screening, with odds ratios (OR) of 0.17, 0.36, and 0.30, respectively^26^. Less likely to have an undated screening were also people 50–54 years old (OR = 0.59 when compared with 70–74 years old), born outside the EU (OR = 0.85), with low education (OR = 0.61), low income (OR = 0.82), and unemployed (OR = 0.88)^26^.

In our study, many survey respondents emphasized adherence challenges. In the free text, several noted that “getting people to actually do the test” is the hardest part. The consensus on using the mail-in FIT for rural areas stems from this practical awareness. Additionally, one point raised was the role of GP endorsement; multiple participants cited evidence that a strong recommendation from a physician is a key driver of patient participation. This reinforces our emphasis on actively involving primary care physicians in the program (from risk assessment to recommendation of screening and follow-up to non-responders).

Moreover, colorectal cancer (CRC) screening initiatives are not only highly cost-effective but also have the potential to save money by reducing the financial strain associated with treating advanced cancer. A study conducted in Australia demonstrated that screening is very cost-effective, with expenses per life-year gained amounting to less than $55,000 annually in 2010 Australian currency^27^. A study conducted in Thailand found that both colonoscopies performed every decade and annual FIT were cost-effective alternatives to no screening. The incremental cost-effectiveness ratios (ICER) for these methods were $600.20 and $509.84 per quality-adjusted life year (QALY) gained, respectively^28^. Colonoscopy demonstrated greater cost-effectiveness compared to FIT, with an ICER of 646.53 USD per QALY gained^28^.

Interestingly, some studies have suggested that CRC screening can be cost-effective and cost-efficient. In Vietnam, the total economic burden of CRC was estimated at $132.9 million in 2018, with indirect costs comprising 83.58% of the total cost^29^. This highlights the potential of screening programs to reduce the economic impact by detecting cancers at earlier stages. Similarly, a Canadian study found that annual FIT screening could save CAN$68 per person over a lifetime compared with no screening^30^.

### Integrated primary care and centralized system approach

The framework integrates primary care physician engagement with centralized program coordination to optimize both accessibility and quality assurance. Primary care physicians serve as the first point of contact for risk assessment, patient education, test distribution, and result communication, leveraging established patient-provider relationships and trust^31^. Simultaneously, a centralized information system manages population registries, invitation scheduling, result tracking, recall protocols, and quality monitoring consistent with successful European models.

### Patient preferences and participation barriers

International studies demonstrate variable patient preferences for colonoscopy versus non-invasive screening methods. Analysis of 1000 participants related to the US Multi-Society Task Force tier 1 tests showed that 68.9% of participants aged 40–49 and 77.4% of people over 50 years old prefer an annual FIT over a colonoscopy each 10 years^32^. A Korean survey revealed that colonoscopy was preferred in 68.7% of cases over FIT (preferred by 31.3% of respondents)^33^. Another evaluation from USA showed a slightly preference for FIT over colonoscopy (0.517 versus 0.483)^34^.

Romanian pilot programs have identified specific barriers including limited health literacy, mistrust of public health initiatives, geographic access challenges in rural areas, and insufficient primary care engagement^11,12^. Successful implementation requires culturally adapted public education campaigns, streamlined test distribution through primary care, and addressing rural-urban healthcare disparities. Evidence suggests that strong physician recommendation is the most powerful predictor of screening participation, reinforcing the framework’s emphasis on primary care physician engagement. Future Romanian programs should incorporate patient preference assessments and barrier analysis to optimize participation across diverse population segments.

### Limitations and future research

Several limitations must be acknowledged. First, the predominance of Romanian professionals (82% in Round 2) may limit international generalizability, though this reflects the study’s focus on developing a Romania-specific framework. Second, the absence of direct patient participation in the consensus process represents a significant limitation; future implementation should incorporate patient preference studies and community engagement. Third, the recommendations lack formal cost-effectiveness analysis specific to Romanian healthcare costs and epidemiology, highlighting the need for economic evaluation studies before national implementation. Fourth, the framework has not undergone pilot testing or validation in Romanian healthcare settings. Finally, the study was conducted during the COVID-19 pandemic, which may have influenced participant perspectives on healthcare system capacity and implementation feasibility. The COVID-19 pandemic disrupted the screening process. Several respondents referenced this: for example, one noted that in their region, “screening colonoscopies were paused for over a year”. Global studies have estimated that pandemic interruptions have led to millions of missed screenings and will likely result in thousands of additional CRC cases and deaths. Our consensus includes planning for catch-up strategies and ensuring the resiliency of screening programs (such as using FIT, which can be performed at home even during pandemics). The survey data showed that participants were acutely aware of these impacts (e.g., 85–95% drops in screening during the peak of the COVID-19 pandemic). On the other hand, the waiting lists for elective diagnostic and therapeutic procedures in symptomatic individuals are increasing in countries around the world, making it challenging to screen asymptomatic patients. An additional challenge for preventive healthcare policies is striking a balance between cancer screening initiatives and addressing other pressing health concerns affecting younger populations such as trauma management and injury prevention.

Despite these limitations, the framework provides evidence-based guidance for Romanian CRC screening program development while acknowledging the need for ongoing refinement based on implementation experience and local data.

The Romanian National Institute of Statistics reported that in 2023 there were 9135391 people over 45 years of age^35^, translating into an estimation of over 180 centers required for CRC screening. Analyses consistently showed that stool-based tests and colonoscopy-based screening strategies fall below the commonly accepted cost-effectiveness thresholds, making organized CRC screening a sound investment for public health systems. However, successful national implementation will require significant investments in healthcare infrastructure, particularly to address the increased demand for colonoscopy and pathology services following positive screening results.

In conclusion, we propose an algorithm for a structured national CRC screening program, a framework that aligns with the country’s demographics, healthcare resources, and logistical realities while maximizing adherence in the target population.

The current approach proposes a coordinated approach for construction and implementation, considering the robust evidence regarding clear and substantial benefits in reducing CRC mortality, increasing the detection of early-stage cancers, and delivering cost-effective healthcare outcomes.

## Methods

Given the pressing need to address CRC screening disparities, we employed a modified Delphi technique to gather professional consensus on effective implementation strategies. The Delphi process consisted of two rounds of anonymous online surveys (SCREECO project: Colorectal cancer SCREEning implementation pathways across developed versus developing healthcare systems: a modified Delphi Consensus, part SCREECO I and SCREECO II) followed by an in-person consensus meeting during the Romanian National Congress of Surgery.

### Literature review strategy

A comprehensive literature review was performed utilizing the PubMed and Google Scholar databases, including the period from January 2010 to December 2021. Search terms included: "colorectal cancer screening," "population screening programs," "fecal immunochemical test," "colonoscopy screening," combined with geographic terms for contexts. Inclusion criteria encompassed: English-language publications, evidence-based screening recommendations, cost-effectiveness analyses, and implementation studies in diverse healthcare systems.

### Panel selection

A multidisciplinary panel was assembled through purposive sampling of the professionals and stakeholders involved in CRC care. The initial questionnaire was developed from the literature review. Invitations to participate in the Delphi survey were disseminated via professional networks, emails, and social media to broaden international input. Although SCREECO is a national project, we sought an international perspective on this topic. We encouraged participation from not only clinicians (surgeons, gastroenterologists, oncologists, and family physicians) but also researchers, health administrators, and patient advocates to capture a wider perspective on screening. Participation was voluntary and the responses were anonymized.

### SCREECO study design and data collection

The SCREECO I and II surveys (see *Appendix B.1 and B.2*) also functioned as cross-sectional studies of current opinions and self-reported practices in CRC screening. In addition to the consensus voting items, the questionnaires collected data on participant’s demographics (age, specialty, years of experience, and country) and contextual information. Notably, we inquired whether the participant’s country had an established national CRC screening program and, if so, how effective they perceived it on a scale of 1 to 10. We also asked open-ended questions in round 1 about the suggested strategies or additional questions for round 2 (captured in Q27). In Round 2, a final open question invited further suggestions for the planned consensus meeting (essentially Round 3 input).

All survey responses were recorded automatically using Google Forms platform. The responses were anonymized prior to the analysis. Participation was voluntary and the study was conducted as a consensus quality improvement initiative without patient health data; therefore, ethics committee approval was not required.

### Delphi round 1 (SCREECO I)

Round 1 used an online questionnaire consisting of two sections. The first section collected general participant data (eight questions on country, specialty, years of experience, etc.) and detailed each participant’s context (e.g., whether their country had a CRC screening program). The second section contained 29 statements or questions related to CRC screening methodology and implementation (e.g., optimal starting age, screening interval, choice of test, and resource allocation). Study participants were instructed to rate their agreement with each provided statement on a Likert scale (typically featuring five points, from Strongly Agree to Strongly Disagree) or to select their preferred options from a set of multiple-choice questions, depending on the specific item. A pilot test of the Round 1 survey was conducted with five respondents to ensure clarity and functionality. Round 1 was open to responses from April 21 to May 15, 2022. We set an a priori consensus threshold such that ≥70% of respondents agreed or strongly agreed with a statement (for Likert-scale items) or selected a given option (for multiple-choice items), which was considered a consensus for round 1. This relatively high threshold was chosen to reflect a strong agreement. Conversely, statements that did not reach 70% agreement were revised or further discussed in round 2.

### Delphi round 2 (SCREECO II)

Round 2 occurred from May 16 to May 31, 2022. A second online questionnaire was distributed to all Round 1 participants who provided contact information, as well as to new participants who wished to join Round 2. Round 2 presented the results of Round 1 and sought consensus on the revised statements. Specifically, any statement from round 1 that failed to meet the 70% consensus threshold was modified based on participant feedback and professional committee input. Some new statements were added in Round 2 to address the topics raised by the respondents in Round 1 (e.g., a proposed screening algorithm synthesizing multiple components). All Round 2 voting was performed using a Likert scale (Agree/Disagree), including confirmation of consensus statements from Round 1 and evaluation of new/revised statements. Participants could also provide free-text comments or suggestions for discussion.

### Consensus meeting

Following the two survey rounds, a dedicated presentation was held during the National Congress of Surgery (June 2022) to review Delphi findings. This served as de facto Round 3, wherein the study coordinators presented the interim results and facilitated discussions among panelists and congress attendees. The outcome of this meeting was a set of final consensus recommendations that were subsequently compiled.

### Statistical analysis

Quantitative data from SCREECO I and II were analyzed using descriptive statistics. After exporting from Google Forms, data cleaning and analysis were performed using statistical software (JAMOVI 2.6.25, and Python 3.13.2 libraries). Artificial Intelligence softwares were used for conceptualization, investigation, review, and editing of the manuscript^36^**.** Categorical variables are summarized as frequencies and percentages. Responses from the Likert scale were considered ordinal data. To analyze continuous variables, we employed non-parametric tests, such as the Mann-Whitney and Kruskal-Wallis methods. For variables in the categorical domain, the chi-squared test was utilized. Statistical significance was declared if P < 0.05.

## Supplementary Information

Supplementary Information.

## Data Availability

The data are available on reasonable request from the corresponding author of the present paper.
