# Correction: Equitable colorectal cancer screening implementation framework via a modified Delphi Consensus for Romanian healthcare

**DOI:** 10.1038/s41598-026-48928-y

**Published:** 2026-05-12

**Authors:** Ionut Negoi, The SCREECO study group, Ionut Negoi, Alexandra Boromiz, Suman Baral, Eduard-Alexandru Bonci, Mihai Botezatu, Teodor Cabel, George Ovidiu Cirstea, Gabriel Constantinescu, Ionut Bogdan Diaconescu, Corneliu Dimitriu, Gabriel Dimofte, Constantin Dina, Florin Dinu, Horia Doran, Sergey Efetov, Biniam Ewnte, Ildar Fakhradiyev, Matteo Frasson, Roxana Ganescu, Dragos Garofil, Zoe Garoufalia, Octav Ginghina, Florin Grama, Claudiu Herteliu, Ioan Tănase, Victor Stefan Ionescu, Hizbullah Jan, Kenneth YY Kok, Sorinel Lunca, Robert Gabriel Lupu, Yasuko Maeda, Iuliana Marin, Radu Mihail Mirica, Alin Moldoveanu, Anca Morar, Francesk Mulita, Ana-Maria Musina, Velenciuc Natalia, Ruxandra Negoi, Omar Rabah Obaid, Akmalbek Otabekov, Francesco Pata, Tapan Patel, Gianluca Pellino, Sorin Stefan Popescu, Xenia Pucheanu, Lupu Robert Gabriel, Luis Rodrigo, Laura Elena Roșu Munteanu, Vasile Sandru, Massimo Sartelli, Haddadi Saïd, Toni Seppälä, Charalampos Seretis, Mostafa Shalaby, Makkai Silviu, Bogdan Stoica, Kire Stojkovski, Marcel Tantau, Ciprian Toma, Elena Adelina Toma, Alexandru-Vicentiu Tudor, Stefana Ilinca Tudorascu, Alexia Ungureanu, Andreea-Catalina Ungureanu, Gabrielle van Ramshorst, Georgios Ioannis Verras, Albina Zubayraeva

**Affiliations:** 1 Carol Davila University of Medicine and Pharmacy Bucharest, Clinical Emergency Hospital of Bucharest, No 8 Floreasca Street, Sector 1, 014461 Bucharest, Romania; 2 Carol Davila University of Medicine and Pharmacy Bucharest, Bucharest, Romania; 3 Dirghayu Pokhara Hospital, Pokhara, Nepal; 4 Iuliu Hatieganu University of Medicine and Pharmacy, Cluj-Napoca, Romania; 5 Clinical Emergency Hospital of Bucharest, Bucharest, Romania; 6 Alexandria County Emergency Hospital, Alexandria, Romania; 7 Jersey General Hospital, Jersey, United Kingdom; 8 Regional Institute of Oncology, University of Medicine and Phramacy Grigore T. Popa, Iasi, Romania; 9 Ovidius University, Constanta, Romania; 10 Public Involvement, Bucharest, Romania; 11 IM Sechenov First Moscow State Medical University, Moscow, Russia; 12 Debre Tabor University, Debre Tabor, Ethiopia; 13 Kazak National Medical University, Almaty, Kazakhstan; 14 University Hospital La Fe, Valencia, Spain; 15 KKH Prignitz, Perleberg, Germany; 16 Cleveland Clinic Florida, Cleveland, Greece; 17 Saint John Emergency Clinical Hospital, Bucharest, Romania; 18 Coltea Clinical Hospital, Bucharest, Romania; 19 Bucharest University of Economic Studies, Bucharest, Romania; 20 Khyber Medical College, Khyber, Pakistan; 21 Pengiran Anak Puteri Rashidah Saadatul Bolkiah Institute of Health Sciences, Universiti Brunei Darussalam, Darussalam, Brunei; 22 Regional Institute of Oncology Iasi, Iasi, Romania; 23 Gheorghe Asachi Technical University of Iasi, Iasi, Romania; 24 Queen Elizabeth University Hospital, Glasgow, United Kingdom; 25 University Politehnica of Bucharest, Bucharest, Romania; 26 Saint John Emergency Hospital Bucharest, Bucharest, Romania; 27 Department of Surgery, General University Hospital of Patras, Patras, Greece; 28 University of Medicine and Pharmacy Grigore T. Popa/Regional Institute of Oncology Iasi, Iasi, Romania; 29 Department of Surgery, Nicola Giannettasio Hospital, Corigliano-Rossano, Corigliano-Rossano, Italy; 30 Baroda Medical College and SSG Hospital, Baroda, India; 31 Department of Advanced Medical and Surgical Sciences, Università degli Studi della Campania Luigi Vanvitelli, Naples, Italy; 32 Zarnesti Hospital, Zarnesti, Romania; 33 HUCA, Oviedo, Spain; 34 Clinical Emergency Hospital of Bucharest, Bucharest, Romania; 35 Macerata Hospital, Macerata, Italy; 36 Central Hospital of the Army, Lucknow, Algeria; 37 University of Tampere/Helsinki, Tampere, Finland; 38 St Andreas General Hospital of Patras, Patras, Greece; 39 Mansoura University, Mansoura, Egypt; 40 Regina Maria Brasov, Brasov, Romania; 41 PSI-CRO, Skopje, Macedonia; 42 University of Medicine and Pharmacy Cluj-Napoca, Cluj-Napoca, Romania; 43 Ghent University Hospital, Ghent, Belgium; 44 General University Hospital of Patras, Patras, Greece

Correction to: *Scientific Reports* 10.1038/s41598-025-26713-7, published online 28 November 2025

The original version of this Article contained an error in Figure 2 and 3, where the two Python scripts used to generate the two figures contain a technical error of the map. The original Figure 2 and 3 and accompanying legend appear below.

The original Article has been corrected.

**Fig. 2:**
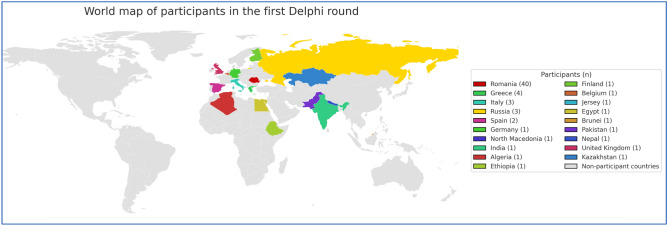
World map of participants in the first survey round.

**Fig. 3:**
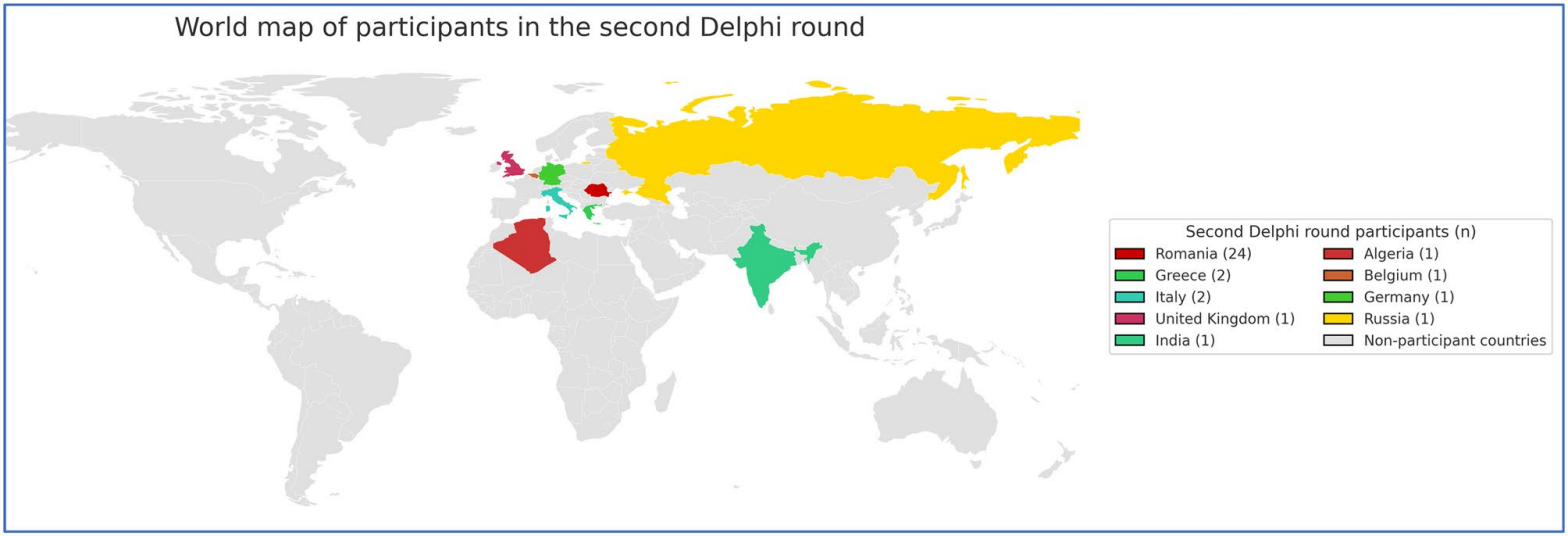
World map of participants in the second survey round.

