# Supplementary material for: Equitable colorectal cancer screening implementation framework via a modified Delphi Consensus for Romanian healthcare
Source: Sci Rep. 2025 Nov 28;15:42692. doi: 10.1038/s41598-025-26713-7 (PMC12663209; doi:10.1038/s41598-025-26713-7)
Supplement: Supplementary file 1 — Supplementary Information. [file 41598_2025_26713_MOESM1_ESM.docx]

Supplementary materials: Equitable Colorectal Cancer Screening Implementation Framework via a Modified Delphi Consensus for Romanian Healthcare

Ionut Negoi ^1,^*, on behalf of the SCREECO study group

^1^*Carol Davila* University of Medicine and Pharmacy Bucharest, Clinical Emergency Hospital of Bucharest, Romania; [ionut.negoi@umfcd.ro](mailto:ionut.negoi@umfcd.ro), [negoiionut@gmail.com](mailto:negoiionut@gmail.com)

***Correspondence**:

**Ionut Negoi M.D. Ph.D.**

Associate Professor of Surgery, *Carol Davila* University of Medicine and Pharmacy Bucharest, Consultant General Surgeon,

Clinical Emergency Hospital of Bucharest, No 8 Floreasca Street, Sector 1, 014461, Bucharest, Romania,

E-mail: [ionut.negoi@umfcd.ro](mailto:ionut.negoi@umfcd.ro), [negoiionut@gmail.com](mailto:negoiionut@gmail.com),

Phone number: +40723209910;

ORCID ID: 0000-0002-6950-9599

Appendix A:

Table A.1 - Results of SCREECO I – First voting round.

Table A.2 Results of the SCREECO II – second voting round

Table A.3 – Review of the published guidelines about colorectal cancer screening.

Appendix B:

Appendix B.1 - Questionnaire for the Round I: SCREECO project: Colorectal cancer SCREEning imple-mentation pathways across developed versus developing healthcare systems: a modified Delphi Consensus

Appendix B.2 - Questionnaire for the Round II: 2nd round SCREECO project: Colorectal cancer SCREEning implementation pathways across developed versus developing healthcare systems: a modified Delphi Consensus: SCREECO project - 2nd ROUND

Appendix C:

Appendix C.1 - Figures of responses from the first voting round

Appendix C.2 - Figures of responses from the second voting round

Appendix A

**Table A.1** Results of SCREECO I – First voting round.

|  | **Overall (N=66)** |
| --- | --- |
| **Country** |  |
| Algeria | 1 (1.5%) |
| Belgium | 1 (1.5%) |
| Brunei | 1 (1.5%) |
| Egypt | 1 (1.5%) |
| Ethiopia | 1 (1.5%) |
| Finland | 1 (1.5%) |
| Germany | 1 (1.5%) |
| Greece | 4 (6.1%) |
| India | 1 (1.5%) |
| Italy | 3 (4.5%) |
| Kazakhstan | 1 (1.5%) |
| Macedonia | 1 (1.5%) |
| Nepal | 1 (1.5%) |
| Pakistan | 1 (1.5%) |
| Romania | 40 (60.6%) |
| Russia | 3 (4.5%) |
| Spain | 2 (3.0%) |
| United Kingdom | 2 (3%) |
| **Age** |  |
| Mean (SD) | 38.4 (11.5) |
| Range | 19.0 - 77.0 |
| **Specialty** |  |
| Gastroenterology | 5 (7.6%) |
| General Population | 2 (3.0%) |
| General Surgery | 41 (62.1%) |
| Medical Imagistics | 1 (1.5%) |
| Medical student | 8 (12.1%) |
| Other | 9 (13.6%) |
| **Hospital type for practice** |  |
| Ambulatory / Office | 1 (1.5%) |
| City Hospital | 11 (16.7%) |
| County Hospital | 1 (1.5%) |
| Others | 3 (4.5%) |
| University / Academic | 50 (75.8%) |
| **Years of clinical experience** |  |
| N-Miss | 10 |
| Mean (SD) | 12.3 (10.4) |
| Range | 0.0 - 45.0 |
| **Does your country have in place a functional national screening programe for CRC?** |  |
| N-Miss | 2 |
| No | 33 (51.6%) |
| Regional not national | 8 (12.5%) |
| Yes | 23 (35.9%) |
| **If YES, how effective / functional do you consider it (from one to ten)?** |  |
| N-Miss | 31 |
| 1 | 1 (2.9%) |
| 2 | 1 (2.9%) |
| 3 | 5 (14.3%) |
| 4 | 6 (17.1%) |
| 5 | 3 (8.6%) |
| 6 | 5 (14.3%) |
| 7 | 4 (11.4%) |
| 8 | 7 (20.0%) |
| 9 | 1 (2.9%) |
| 10 | 2 (5.7%) |
| **Q 1: General Practitioner should include at the patients first visit an assessment if the patient is at increased risk for colorectal cancer** |  |
| No | 1 (1.5%) |
| Others | 1 (1.5%) |
| Yes | 64 (97.0%) |
| **Q 2: General Practitioner should evaluate the risks to develop a CCR starting from the patients age of:** |  |
| 20 | 14 (21.2%) |
| 25 | 6 (9.1%) |
| 30 | 6 (9.1%) |
| 35 | 40 (60.6%) |
| **Q 3: General practitioner should reassess the risk of patients to develop a CCR at an interval of:** |  |
| 3 years | 36 (54.5%) |
| 5 years | 27 (40.9%) |
| Other | 3 (4.5%) |
| **Q 4: General Practitioner Visit assessment if the patients is at increased risk for colorectal cancer should include as standard:** |  |
| Familial history of cancer or adenomatous polyps | 18 (26.3%) |
| Familial history of cancer or adenomatous polyps, Familial history of genetic syndromes associated with CRC, Personal history of ulcerative colitis or Crohn disease | 2 (3.0%) |
| Familial history of cancer or adenomatous polyps, Personal history of CRC or benign colorectal pathology such as adenomatous polyps, Familial history of genetic syndromes associated with CRC | 2 (3.0%) |
| Familial history of cancer or adenomatous polyps, Personal history of CRC or benign colorectal pathology such as adenomatous polyps, Familial history of genetic syndromes associated with CRC, Personal history of ulcerative colitis or Crohn disease | 5 (7.6%) |
| Familial history of cancer or adenomatous polyps, Personal history of CRC or benign colorectal pathology such as adenomatous polyps, Familial history of genetic syndromes associated with CRC, Personal history of ulcerative colitis or Crohn disease, Personal history of abdominal radiation for pediatric cancers | 19 (28.8%) |
| Familial history of cancer or adenomatous polyps, Personal history of CRC or benign colorectal pathology such as adenomatous polyps, Personal history of abdominal radiation for pediatric cancers | 1 (1.5%) |
| Familial history of genetic syndromes associated with CRC | 9 (13.6%) |
| Familial history of genetic syndromes associated with CRC (Familial adenomatous polyposis, Lynch syndrome, etc.) | 1 (1.5%) |
| Personal history of CRC or benign colorectal pathology such as adenomatous polyps | 6 (9.1%) |
| Personal history of CRC or benign colorectal pathology such as adenomatous polyps, Familial history of genetic syndromes associated with CRC | 1 (1.5%) |
| Personal history of CRC or benign colorectal pathology such as adenomatous polyps, Familial history of genetic syndromes associated with CRC, Personal history of ulcerative colitis or Crohn disease | 1 (1.5%) |
| Personal history of abdominal radiation for pediatric cancers | 1 (1.5%) |
| **Q 5: Patients with no risk factors from the above question fall in the average risk population** |  |
| Agree | 50 (75.8%) |
| Disagree | 16 (24.2%) |
| **Q 7: Age for starting screening in average risk population** |  |
| 40 | 16 (24.2%) |
| 45 | 21 (31.8%) |
| 50 | 26 (39.4%) |
| 55 | 3 (4.5%) |
| **Q 8: Do you recommend as frequency for the screening in AVERAGE risk population:** |  |
| Each five years | 35 (53.0%) |
| Each ten years | 7 (10.6%) |
| Each two years | 19 (28.8%) |
| Each year | 5 (7.6%) |
| **Q 9: Age for starting screening in high risk population** |  |
| 20 | 15 (22.7%) |
| 25 | 8 (12.1%) |
| 30 | 11 (16.7%) |
| 35 | 16 (24.2%) |
| 40 | 12 (18.2%) |
| 45 | 1 (1.5%) |
| 50 | 3 (4.5%) |
| **Q 10: Do you recommend as frequency for the screening in HIGH risk population:** |  |
| Each five years | 5 (7.6%) |
| Each ten years | 1 (1.5%) |
| Each two years | 29 (43.9%) |
| Each year | 31 (47.0%) |
| **Q 11: Screening discontinuation should be at the age of:** |  |
| 70 | 11 (16.7%) |
| 75 | 19 (28.8%) |
| 80 | 18 (27.3%) |
| 85 | 18 (27.3%) |
| **Q 12: Screening methods which maybe considered for a national implementation** |  |
| Colonoscopy | 6 (9.1%) |
| Colonoscopy, Computed Tomography colonography | 2 (3.0%) |
| Colonoscopy, Sigmoidoscopy | 2 (3.0%) |
| Colonoscopy, Sigmoidoscopy, Computed Tomography colonography | 1 (1.5%) |
| Fecal immunochemical tests (FIT) | 13 (19.7%) |
| Fecal immunochemical tests (FIT) , Colonoscopy | 1 (1.5%) |
| Fecal immunochemical tests (FIT), Colonoscopy | 12 (18.2%) |
| Fecal immunochemical tests (FIT), Colonoscopy, Computed Tomography colonography | 5 (7.6%) |
| Fecal immunochemical tests (FIT), Guaiac-based fecal occult tests | 5 (7.6%) |
| Fecal immunochemical tests (FIT), Guaiac-based fecal occult tests, Colonoscopy | 6 (9.1%) |
| Fecal immunochemical tests (FIT), Guaiac-based fecal occult tests, Colonoscopy, Sigmoidoscopy, Computed Tomography colonography | 7 (10.6%) |
| Fecal immunochemical tests (FIT), Guaiac-based fecal occult tests, Sigmoidoscopy | 2 (3.0%) |
| Fecal immunochemical tests (FIT), Sigmoidoscopy | 2 (3.0%) |
| Guaiac-based fecal occult tests, Colonoscopy | 1 (1.5%) |
| Guaiac-based fecal occult tests, Colonoscopy, Sigmoidoscopy | 1 (1.5%) |
| **Q 13: Estimated sensitivity for colorectal cancer of the following screening methods is: [Fecal immunochemical tests every year (FIT)]** |  |
| N-Miss | 5 |
| 70%-75% | 17 (27.9%) |
| 75%-80% | 23 (37.7%) |
| 80%-85% | 11 (18.0%) |
| 85%-90% | 4 (6.6%) |
| 90%-95% | 3 (4.9%) |
| 95%-100% | 3 (4.9%) |
| **Q 13: Estimated sensitivity for colorectal cancer of the following screening methods is: [Guaiac-based fecal occult tests every year]** |  |
| N-Miss | 7 |
| 70%-75% | 23 (39.0%) |
| 75%-80% | 20 (33.9%) |
| 80%-85% | 12 (20.3%) |
| 85%-90% | 2 (3.4%) |
| 90%-95% | 1 (1.7%) |
| 95%-100% | 1 (1.7%) |
| **Q 13: Estimated sensitivity for colorectal cancer of the following screening methods is: [Colonoscopy every 10 years]** |  |
| N-Miss | 4 |
| 70%-75% | 12 (19.4%) |
| 75%-80% | 7 (11.3%) |
| 80%-85% | 14 (22.6%) |
| 85%-90% | 7 (11.3%) |
| 90%-95% | 14 (22.6%) |
| 95%-100% | 8 (12.9%) |
| **Q 13: Estimated sensitivity for colorectal cancer of the following screening methods is: [Sigmoidoscopy every 5 years]** |  |
| N-Miss | 6 |
| 70%-75% | 13 (21.7%) |
| 75%-80% | 14 (23.3%) |
| 80%-85% | 12 (20.0%) |
| 85%-90% | 9 (15.0%) |
| 90%-95% | 9 (15.0%) |
| 95%-100% | 3 (5.0%) |
| **Q 13: Estimated sensitivity for colorectal cancer of the following screening methods is: [Computed Tomography colonography ]** |  |
| N-Miss | 6 |
| 70%-75% | 8 (13.3%) |
| 75%-80% | 14 (23.3%) |
| 80%-85% | 12 (20.0%) |
| 85%-90% | 7 (11.7%) |
| 90%-95% | 12 (20.0%) |
| 95%-100% | 7 (11.7%) |
| **Q 14: Estimated prevented death due to colorectal cancer per 1000 people older than 40 years [Fecal immunochemical tests every year (FIT)]** |  |
| N-Miss | 7 |
| 10-15 | 19 (32.2%) |
| 15-20 | 17 (28.8%) |
| 20-25 | 14 (23.7%) |
| 25-30 | 9 (15.3%) |
| **Q 14: Estimated prevented death due to colorectal cancer per 1000 people older than 40 years [Guaiac-based fecal occult tests every year]** |  |
| N-Miss | 9 |
| 10-15 | 20 (35.1%) |
| 15-20 | 24 (42.1%) |
| 20-25 | 9 (15.8%) |
| 25-30 | 4 (7.0%) |
| **Q 14: Estimated prevented death due to colorectal cancer per 1000 people older than 40 years [Colonoscopy every 10 years]** |  |
| N-Miss | 6 |
| 10-15 | 8 (13.3%) |
| 15-20 | 6 (10.0%) |
| 20-25 | 32 (53.3%) |
| 25-30 | 14 (23.3%) |
| **Q 14: Estimated prevented death due to colorectal cancer per 1000 people older than 40 years [Sigmoidoscopy every 5 years]** |  |
| N-Miss | 9 |
| 10-15 | 9 (15.8%) |
| 15-20 | 16 (28.1%) |
| 20-25 | 19 (33.3%) |
| 25-30 | 13 (22.8%) |
| **Q 14: Estimated prevented death due to colorectal cancer per 1000 people older than 40 years [Computed Tomography colonography every 5 years]** |  |
| N-Miss | 8 |
| 10-15 | 14 (24.1%) |
| 15-20 | 10 (17.2%) |
| 20-25 | 18 (31.0%) |
| 25-30 | 16 (27.6%) |
| **Q 15: What screening strategy will you choose for you as a patient:** |  |
| Colonoscopy every 10 years | 24 (36.4%) |
| Fecal immunochemical tests every year / CT colonography every 5 years THAN colonoscopy in the case of a positive initial test | 33 (50.0%) |
| Others (sigmoidoscopy with fecal immunochemical tests OR sigmoidoscopy alone OR capsule colonoscopy) | 9 (13.6%) |
| **Q 16: What screening strategy will you consider with a maximum of adherence for patients from urban areas:** |  |
| Colonoscopy every 10 years | 20 (30.3%) |
| Colonoscopy every 10 years (Colonoscopie la fiecare 10 ani) | 1 (1.5%) |
| Fecal immunochemical tests every year / CT colonography every 5 years THAN colonoscopy in the case of a positive initial test | 36 (54.5%) |
| Others (sigmoidoscopy with fecal immunochemical tests OR sigmoidoscopy alone OR capsule colonoscopy) | 8 (12.1%) |
| Others (sigmoidoscopy with fecal immunochemical tests OR sigmoidoscopy alone OR capsule colonoscopy) - Alte metode (sigmoidoscopie combinată cu test fecal imunochimic SAU sigmoidoscopie SAU colonoscopie prin capsulă) | 1 (1.5%) |
| **Q 17: What screening strategy will you consider with a maximum of adherence for patients from rural areas:** |  |
| Colonoscopy every 10 years | 17 (25.7%) |
| Fecal immunochemical tests every year / CT colonography every 5 years THEN colonoscopy in the case of a positive initial test | 41 (62.1%) |
| Others (sigmoidoscopy with fecal immunochemical tests OR sigmoidoscopy alone OR capsule colonoscopy) | 7 (10.6%) |
| Others (sigmoidoscopy with fecal immunochemical tests OR sigmoidoscopy alone OR capsule colonoscopy) | 1 (1.5%) |
| **Q 18: What screening strategy will you choose in case of limited acces to colonoscopy:** |  |
| CT colonography every 5 years | 12 (18.2%) |
| Capsule colonoscopy | 2 (3.0%) |
| Capsule colonoscopy - colonoscopie prin capsulă | 1 (1.5%) |
| Fecal immunochemical tests every year / THEN colonoscopy in the case of a positive initial test | 28 (42.4%) |
| Guaiac-based fecal occult tests every year | 7 (10.6%) |
| Sigmoidoscopy alone | 1 (1.5%) |
| Sigmoidoscopy with fecal immunochemical tests | 14 (21.2%) |
| Sigmoidoscopy with fecal immunochemical tests - Sigmoidoscopie combinată cu test fecal imunochimic | 1 (1.5%) |
| **Q 19: In high risk patients what screening strategy will you choose for you as a patient: [Colonoscopy]** |  |
| N-Miss | 3 |
| Every three years | 15 (23.8%) |
| Every two year | 24 (38.1%) |
| Every year | 24 (38.1%) |
| **Q 19: In high risk patients what screening strategy will you choose for you as a patient: [Fecal immunochemical tests / CT colonography THAN colonoscopy in the case of a positive initial test]** |  |
| N-Miss | 6 |
| Every three years | 10 (16.7%) |
| Every two year | 10 (16.7%) |
| Every year | 40 (66.7%) |
| **Q 19: In high risk patients what screening strategy will you choose for you as a patient: [Others (sigmoidoscopy with fecal immunochemical tests OR sigmoidoscopy alone OR capsule colonoscopy) ]** |  |
| N-Miss | 13 |
| Every three years | 10 (18.9%) |
| Every two year | 21 (39.6%) |
| Every year | 22 (41.5%) |
| **Q 20: What costs do you think are for a colonoscopy (all hidden costs, including single use medical devices, human resources, service and technologies depreciation, etc.)?** |  |
| N-Miss | 12 |
| Mean (SD) | 260.2 (89.2) |
| Range | 100.0 - 400.0 |
| **Q 21: Do you consider public sector reimbursement for colonoscopy to cover all the costs?** |  |
| No | 17 (25.8%) |
| Yes | 49 (74.2%) |
| **Q 22: Do you consider that reimbursement from public sector of all the costs for screening colonoscopy and early treatment will decrease the costs for healthcare system (decreasing the costs for medical care for patients with advanced disease, decreasing the burden of the disease in the society, increasing the contribution for society of treated patients, etc)?** |  |
| No | 4 (6.1%) |
| Yes | 62 (93.9%) |
| **Q 23: Do you consider for your country to start screening directly with colonoscopy in high risk population, as defined by general practitioner?** |  |
| No | 5 (7.6%) |
| Other options | 5 (7.6%) |
| Yes | 56 (84.8%) |
| **Q 24: Do you agree that all hospitals / available resources should be involved in screening?** |  |
| No | 8 (12.1%) |
| Yes | 58 (87.9%) |
| **Q 25: What number of general populations should be allocated to a screening unit (human and material resources dedicated to screening in a hospital)?** |  |
| 100 | 2 (3.0%) |
| 100 000 | 1 (1.5%) |
| 100/year | 1 (1.5%) |
| 1000 | 4 (6.1%) |
| 10000 | 8 (12.1%) |
| 100000 | 6 (9.1%) |
| 15000 | 2 (3.0%) |
| 2 Per week | 1 (1.5%) |
| 20 | 1 (1.5%) |
| 200 | 1 (1.5%) |
| 2000 | 2 (3.0%) |
| 20000 | 3 (4.5%) |
| 200000 | 1 (1.5%) |
| 25000 | 1 (1.5%) |
| 30000 | 1 (1.5%) |
| 3500 | 1 (1.5%) |
| 40,000-50,000 | 1 (1.5%) |
| 500 | 1 (1.5%) |
| 500-1000 | 1 (1.5%) |
| 5000 | 3 (4.5%) |
| 50000 | 1 (1.5%) |
| 60 | 1 (1.5%) |
| 6000 | 1 (1.5%) |
| 75000 | 1 (1.5%) |

**Table A.2** Results of the SCREECO II – second voting round

|  | **Overall (N=34)** |
| --- | --- |
| **Specialty** |  |
| Gastroenterology | 3 (8.8%) |
| General Population | 2 (5.9%) |
| General Surgery | 23 (67.6%) |
| Medical Imagistics | 1 (2.9%) |
| Medical student | 1 (2.9%) |
| Other | 4 (11.8%) |
| **Hospital type for practice** |  |
| Ambulatory / Office | 1 (2.9%) |
| City Hospital | 5 (14.7%) |
| County Hospital | 2 (5.9%) |
| Others | 2 (5.9%) |
| University / Academic | 24 (70.6%) |
| **General Practitioner should evaluate the risks to develop a CCR starting from the patients age of 20-25 years old.** |  |
| Agree | 10 (29.4%) |
| Disagree | 15 (44.1%) |
| Neutral | 2 (5.9%) |
| Strongly Disagree | 1 (2.9%) |
| Strongly agree | 6 (17.6%) |
| **General practitioner should reassess the risk of patients to develop a CCR at an interval of 3 (three) years.** |  |
| Agree | 15 (44.1%) |
| Disagree | 5 (14.7%) |
| Neutral | 3 (8.8%) |
| Strongly Disagree | 1 (2.9%) |
| Strongly agree | 10 (29.4%) |
| **General Practitioner assessment if the patients is at increased risk for colorectal cancer should include as standard the following: (1) Familial history of cancer or adenomatous polyps; (2) Personal history of CRC or benign colorectal pathology such as adenomatous polyps; (3) Familial history of genetic syndromes associated with CRC; (4) Personal history of ulcerative colitis or Crohn disease; (5) Personal history of abdominal radiation for pediatric cancers.** |  |
| Agree | 16 (47.1%) |
| Neutral | 1 (2.9%) |
| Strongly agree | 17 (50.0%) |
| **Age for starting screening in average risk population should be 45 years old.** |  |
| Agree | 19 (55.9%) |
| Disagree | 2 (5.9%) |
| Neutral | 2 (5.9%) |
| Strongly agree | 11 (32.4%) |
| **Screening discontinuation should be at the age of 75 years old.** |  |
| Agree | 11 (32.4%) |
| Disagree | 6 (17.6%) |
| Neutral | 7 (20.6%) |
| Strongly agree | 10 (29.4%) |
| **Do you agree with the below pathways, divided for urban (better- or less-informed) and rural (high- and low-population) areas ?** |  |
| Agree | 22 (64.7%) |
| Disagree | 1 (2.9%) |
| Neutral | 4 (11.8%) |
| Strongly agree | 7 (20.6%) |

Table A.3 – Review of the published guidelines about colorectal cancer screening.

| Title, year of publication, reference, region | Starting age for screening | Discontinuation age for screening | Methods of screening | Observations |
| --- | --- | --- | --- | --- |
| Clinical practice guidelines for the prevention, early detection and management of colorectal cancer - Cancer Council Australia – 2017 ^1^, Australia | 50 years for average-risk individuals; 25 years for high-risk individuals such as those with Lynch Syndrome | 74 years | FIT (iFOBT) and colonoscopy are used for colorectal cancer screening. | A revised presentation of clinical guidelines for healthcare professionals in Australia, focusing on the prevention, early identification, and treatment of colorectal cancer. |
| Clinical practice guidelines for the prevention, early detection, and management of colorectal cancer: Population screening – 2018 ^2^, Australia | 45 years | 74 years (routine discontinuation age), with exceptions for individuals aged 75-85 years based on health status and personal request | - FIT (iFOBT): every two years  - Flexible sigmoidoscopy: Not recommended  - Colonoscopy: Follow-up after positive iFOBT, not primary screening | The document outlines recommendations for colorectal cancer screening in individuals with average risk between the ages of 45 and 74. It suggests using immunochemical fecal occult blood tests every two years, while stressing the importance of fair access and high-quality follow-up procedures..  Biennial screening using an immunochemical fecal occult blood test (iFOBT) for people between 45 and 74 years old.Biennial iFOBT screening may be recommended for people in the age groups of 75-85 and 40-44 years, subject to certain requirements.  - Sensitivity of iFOBT: 92% (95% CI 74-98)  - Specificity of iFOBT: 88% (95% CI 86-90)  - Yearly iFOBT vs. 2-yearly iFOBT:    - CRC incidence reduction: 9-10% at 40% participation, 21-22% at 100% participation    - CRC mortality reduction: 15% at 40% participation, 26-29% at 100% participation  - Five-yearly stool biomarker:    - Sensitivity for CRC: 85.7%-92.9%    - Specificity for CRC: 84.9%-88.5%    - Sensitivity for advanced adenoma: 47.8%  - Cost-effectiveness:    - 2-yearly iFOBT is cost-saving compared to no screening    - Five-yearly stool biomarker is more expensive and less effective compared to 2-yearly iFOBT |
| Clinical Practice Guideline on Screening for Colorectal Cancer in Individuals With a Family History of Nonhereditary Colorectal Cancer or Adenoma: The Canadian Association of Gastroenterology Banff Consensus – 2018 ^3^, Canada and United States | 40-50 years or 10 years younger than the age of diagnosis of the FDR, whichever is earlier, for those with a family history; age 50 for average-risk individuals. | - | - Colonoscopy: every 5 years for individuals with 2 or more FDRs with CRC; every 5-10 years for individuals with 1 FDR with CRC or advanced adenoma.  - FIT: every 1-2 years as an alternative to colonoscopy. | Screening Recommendations for Colorectal Cancer in Individuals with Familial Risk Factors:  1. Individuals with two or more first-degree relatives (FDRs) diagnosed with colorectal cancer (CRC) should undergo screening colonoscopy every five years, starting at age 40–50 or ten years before the earliest family diagnosis, whichever occurs first.  2. Individuals with one first-degree relative diagnosed with CRC or advanced adenoma should initiate colonoscopy screening every five years, beginning at age 40–50 years or ten years earlier than the relative’s age at diagnosis, whichever occurs first.  Risk Estimates Associated with Family History of Colorectal Cancer:  - Individuals with one first-degree relative diagnosed with colorectal cancer have a 31% increased relative risk (RR = 1.31, 95% CI, 1.11-1.55).  - Individuals with a second-degree relative diagnosed with colorectal cancer demonstrate an elevated risk, but the association was statistically non-significant (RR = 6.11, 95% CI, 0.38-98.00).  - Individuals with two or more first-degree relatives affected by colorectal cancer have a significantly elevated pooled relative risk of 5.77 (95% CI, 3.3-10.1).  - The relative risk for colorectal cancer among individuals with a family history involving one first-degree relative is approximately doubled (RR ranging from 1.79 to 3.55), with higher risks observed when relatives were diagnosed at younger ages.  - The presence of advanced adenomas in siblings significantly increases the odds of developing advanced adenomas (OR = 6.05; 95% CI, 2.74-13.36) or any adenomas (OR = 3.29, 95% CI, 2.16-5.03).  - Individuals with second-degree relatives diagnosed with colorectal cancer and at least one affected first-degree relative also experience increased colorectal cancer risk (RR = 2.37; 95% CI, 1.92-2.92; P = .03). |
| ACG Clinical Guidelines: Colorectal Cancer Screening - 2021^4^, United States | 45 years for conditional recommendation; 50 years for strong recommendation | 86 years | - gFOBT: Biennial (every two years)  - FIT: Every year  - Sigmoidoscopy: Every 5–10 years  - Colonoscopy: Every 10 years  - Computed Tomography (CTC): Every 5 years | The updated American College of Gastroenterology guidelines provide comprehensive recommendations for colorectal cancer (CRC) screening in average-risk individuals and those with familial risk factors. Additionally, these guidelines address adjunctive measures such as aspirin chemoprevention, colonoscopy quality standards, and strategies aimed at enhancing patient adherence. The ultimate goal is to improve screening effectiveness, thereby reducing CRC incidence and associated mortality.  Recommended Screening Modalities:  1. Colonoscopy: every 10 years.  2. Fecal Immunochemical Test (FIT): annually.  3. Multitarget Stool DNA Test: every 3 years.  4. Flexible Sigmoidoscopy: every 5 to 10 years.  5. Computed Tomographic Colonography (CT colonography): every 5 years.  6. Capsule Colonoscopy: every 5 years.  7. Low-dose Aspirin: recommended selectively for individuals aged 50–69 years who have a ≥10% 10-year cardiovascular risk, but not as an alternative to CRC screening.  Performance and Impact of Screening Tests:  -Colonoscopy:  - Reduction in CRC incidence: approximately 69%.  - Reduction in CRC mortality: approximately 68%.  Flexible Sigmoidoscopy:  - Reduction in CRC incidence: approximately 20%.  - Reduction in CRC mortality: approximately 27%.  Fecal Immunochemical Test (FIT):  - Sensitivity: 79%–91%.  - Specificity: 90%–94%.  Multitarget Stool DNA Test:  - Sensitivity for CRC detection: approximately 92%.  - Specificity for CRC detection: approximately 87%.  Computed Tomographic Colonography (CTC):  - Recommended interval: every 5 years.  - Sensitivity and specificity vary depending on lesion size, with high accuracy for lesions ≥10 mm.  Colon Capsule Endoscopy:  - Sensitivity for adenomas ≥6 mm: approximately 81%.  - Specificity: approximately 93%.  Methylated Septin 9 Blood Test:  - Sensitivity for CRC detection: approximately 48%.  FIT:  - Sensitivity for CRC detection ranges from 79% to 91%.  - Specificity ranges from 90% to 94%. |
| Part 1: Screening for the Purposes of Colorectal Cancer Prevention and Detection in Asymptomatic Adults – 2022 ^5^, British Columbia, Canada | 50 years for average-risk individuals | 75 years, with a final cutoff at 85 years | - FIT: every 2 years  - Colonoscopy: every 5 years for high-risk individuals, every 10 years for average-risk individuals  - Flexible Sigmoidoscopy: every 10 years  - CT Colonography: every 5 years | Recommended Screening Protocols:  1. Fecal Immunochemical Testing (FIT): Recommended biennially for average-risk individuals aged 50 to 74 years.  2. Colonoscopy: Recommended every five years for individuals with a significant family history of colorectal cancer, initiating at age 40 or at least 10 years before the youngest affected relative’s diagnosis.  3. Diagnostic Colonoscopy: Indicated following any positive FIT result.  5. Flexible Sigmoidoscopy: Every 10 years, as an alternative modality for average-risk screening.  5. Computed Tomographic Colonography (CTC): Every 5 years, offered as an alternative for average-risk individuals. |
| Colorectal Cancer Screening (PDQ®)–Health Professional Version – 2024 ^6^, United States, Poland, Canada, Germany | 50 years | 75 years  After 75 – individualized | - gFOBT: Not specified  - FIT: Not specified  - Sigmoidoscopy: Not specified  - Colonoscopy: Every 10 years  - Computed Tomography: Not specified | The paper provides a comprehensive overview of colorectal cancer screening, discussing its prevalence, risk factors, effectiveness of various screening methods, and associated potential harms, while emphasizing the importance of regular updates to incorporate new evidence.  1. Colonoscopy  2. Flexible sigmoidoscopy  3. Fecal Occult Blood Test (guaiac-based)  4. Fecal Occult Blood Test (fecal immunochemical-based: FIT)  5. Virtual colonoscopy (CTC or CT pneumocolon)  6. cfDNA blood test  - Incidence rate decline: 1% per year from 2011 to 2019  - Mortality rate decline: 1.8% per year over the last decade  - Danish trial CRC mortality reduction:    - 18% at 10 years    - 15% at 13 years (RR, 0.85; 95% CI, 0.73-1.00)    - 11% at 17 years (RR, 0.89; 95% CI, 0.78-1.01)  - Polish Colonoscopy Screening Program:    - Standardized incidence ratio:      - LQC: 0.32 (95% CI, 0.29-0.35)      - HQC: 0.16 (95% CI, 0.13-0.20)    - Standardized mortality ratio:      - LQC: 0.22 (95% CI, 0.18-0.25)      - HQC: 0.10 (95% CI, 0.06-0.14)  - Multitargeted stool DNA test sensitivity for CRC: 92.3%  - FIT sensitivity for CRC: 73.8%  - Multitargeted stool DNA test sensitivity for advanced lesions: 42.4%  - FIT sensitivity for advanced lesions: 23.8%  - cfDNA blood-based test sensitivity for CRC: 83.1% |
| Canadian Association of Gastroenterology position statement on screening individuals at average risk for developing colorectal cancer: 2010 ^7^, Canada | 50 years | 75 years | - gFOBT every year or every two years  - FIT every year  - Sigmoidoscopy every 10 years  - Colonoscopy every 10 years  - CT every 5 years | The Canadian guidelines emphasize structured colorectal cancer (CRC) screening protocols tailored for average-risk individuals, advocating evidence-based modalities including fecal occult blood testing (FOBT) and flexible sigmoidoscopy (FS). They also set clear recommendations regarding the upper age threshold for discontinuing routine CRC screening.  Recommended Screening Strategies:  1. Fecal Occult Blood Testing (FOBT) performed annually or biennially.  2. Flexible Sigmoidoscopy (FS) routinely recommended for individuals at average risk.  3. Colonoscopy recommended selectively for opportunistic screening.  Effectiveness of Recommended Screening Methods:  • Fecal Occult Blood Testing (FOBT): Biennial or annual testing schedules.  • Annual FIT screening increases life-years gained from 198 to 227 per 1000 individuals screened.  • Fecal Immunochemical Test (FIT):  • Annual FIT shows substantial sensitivity and has demonstrated effectiveness in increasing life-years gained, as noted above.  • Flexible Sigmoidoscopy:  • Reduces CRC incidence by 23% (intention-to-treat) to 33% (per-protocol analysis).  • Reduces CRC-related mortality by 23% (intention-to-treat) to 43% (per-protocol analysis).  • Adherence and Opportunistic Screening:  • Colonoscopy remains an acceptable method for opportunistic screening scenarios outside structured programs. |
| Colorectal Cancer Screening for Average-Risk Adults: 2018 Guideline Update From the American Cancer Society – 2018 ^8^, United States | 45 years | 75-85 years | - gFOBT: every year  - FIT: every year  - Sigmoidoscopy: every 5 years  - Colonoscopy: every 10 years  - Computed Tomography (CT colonography): every 5 years | The updated colorectal cancer (CRC) screening guidelines issued by the American Cancer Society provide comprehensive recommendations for average-risk individuals beginning at 45 years of age. The guidelines emphasize multiple screening methods, allowing patient choice, and individualized approaches to enhance compliance, early detection, and survival outcomes.  Recommended Screening Modalities and Intervals:  1. Annual fecal immunochemical test (FIT).  2. Annual high-sensitivity guaiac-based fecal occult blood test (HSgFOBT).  3. Multitarget stool DNA testing (mt-sDNA) every 3 years.  4. Colonoscopy every 10 years.  5. Computed tomography colonography (CTC) every 5 years.  6. Flexible sigmoidoscopy every 5 years.  Estimated Life-Years Gained per 1,000 Individuals Screened:  Colonoscopy (every 10 years):  • Ages 45-75: 429 life-years  • Ages 50-75: 404 life-years  Computed tomography colonography every 5 years:  • 390 life-years (45-75 years)  • 368 life-years (50-75 years)  Flexible sigmoidoscopy every 5 years:  • 403 life-years (45-75 years)  • 380 life-years (50-75 years)  Annual high-sensitivity guaiac-based fecal occult blood test (HSgFOBT):  • 403 life-years (45-75 years)  • 377 life-years (50-75 years)  Annual fecal immunochemical test (FIT):  • 403 life-years (45-75 years)  • 377 life-years (50-75 years)  Multitarget stool DNA test every 3 years:  • 376 life-years (45-75 years)  • 350 life-years (50-75 years)  Procedure Utilization (per 1000 screened individuals):  Colonoscopy every 10 years:  • 5646 procedures (ages 45-75 years)  • 4836 procedures (50-75 years)  Computed tomography colonography every 5 years:  • 2666 procedures (45-75 years)  • 2430 procedures (50-75 years)  Flexible sigmoidoscopy every 5 years:  • 3761 procedures (45-75 years)  • 3426 procedures (50-75 years)  Annual FIT:  • 2698 colonoscopies (45-75 years)  • 2402 colonoscopies (50-75 years) |
| Canadian Task Force on Preventive Health Care: Screening for Colorectal Cancer -Clinician Summary -2016 ^9,10^, Canada | 50 years | 75 years | - gFOBT: every two years  - FIT: every two years  - Sigmoidoscopy: every 10 years  - Colonoscopy: Not recommended  - Computed Tomography: Not mentioned | The paper provides recommendations for colorectal cancer screening in adults aged 50 and over, emphasizing different strategies based on age groups, with strong recommendations for ages 60-74, weak recommendations for ages 50-59, and advising against screening for those aged 75 and over, while also recommending against routine use of colonoscopy due to lack of direct evidence.  - FOBT (either gFOBT or FIT) every two years for adults aged 50 to 74.  - Flexible sigmoidoscopy every 10 years for adults aged 50 to 74. |
| Scottish Intercollegiate Guidelines Network: Diagnosis and management of colorectal cancer - A national clinical guideline – 2016 ^11^, Scotland | 50 years | 75 years | - gFOBT: Not specified  - FIT: Used for population screening, no specific interval mentioned  - Sigmoidoscopy: Not mentioned  - Colonoscopy: Recommended for high-risk groups (e.g., after 10 years of ulcerative colitis or Crohn's colitis), intervals determined by risk stratification | The updated SIGN guidelines offer comprehensive colorectal cancer (CRC) recommendations based on current evidence, emphasizing interventions aimed at improving patient outcomes, survival rates, and quality of life. These guidelines provide clear clinical pathways for CRC prevention, detection, and treatment.  Highlighted Interventions and Recommendations:  • Screening modalities: Colonoscopy, computed tomography (CT) colonography, and guaiac-based fecal occult blood testing (FOBT).  • Surgical treatment: Total mesorectal excision for rectal cancer.  • Adjuvant therapy: Chemotherapy recommended for Stage III colorectal cancer.  • Diagnostic imaging: Magnetic resonance imaging (MRI) for accurate staging.  • Multidisciplinary approach: Routine multidisciplinary team discussions to optimize treatment planning.  • Post-treatment follow-up: Structured surveillance protocols after curative-intent surgery.  Screening Test Performance and Outcomes:  • Guaiac-based FOBT significantly reduces CRC mortality, particularly when adherence is high.  • Fecal immunochemical testing (FIT):  • Demonstrates sensitivity, specificity, positivity rate, and positive predictive value comparable to guaiac FOBT.  • Achieves greater patient participation and lower procedural errors compared with guaiac-based testing.  • Results in increased colonoscopy follow-up rates due to higher test positivity compared to guaiac FOBT. |
| Screening for Colorectal Cancer in Asymptomatic Average-Risk Adults: A Guidance Statement From the American College of Physicians (Version 2) – 2023 ^12^, United States | 50 years | 75 years | gFOBT, FIT, CTC; specific intervals not mentioned | The paper provides updated guidance for clinicians on colorectal cancer screening in asymptomatic average-risk adults, based on the USPSTF's 2021 evidence review and decision modeling, discussing various screening methods and their effectiveness.  gFOBT (guaiac-based fecal occult blood test), FIT (fecal immunochemical test)  - FIT (Fecal Immunochemical Test):    - Sensitivity: 0.74 (CI, 0.64 to 0.83)    - Specificity: 0.94 (CI, 0.93 to 0.96)    - Modeling study specificity: 0.97  - gFOBT (guaiac-based fecal occult blood test):    - No differences in all-cause mortality found in four RCTs  - sDNA tests and CTC:    - No eligible studies evaluating effectiveness for CRC screening |
| American College of Radiology  ACR Appropriateness Criteria®  Colorectal Cancer Screening – 2018 ^13^, United States | 45 years | 75 years | Colorectal cancer screening utilizes CT colonography (CTC) without intravenous contrast. The screening protocol consists of an initial examination, and if the results are negative, subsequent scans are performed at 5-year intervals. Other methods like gFOBT, FIT, sigmoidoscopy, and colonoscopy are not covered in this document. | The current colorectal cancer screening guidelines highlight the utility of computed tomographic colonography (CTC) without intravenous contrast as an effective modality for screening average-risk and select elevated-risk adults aged 45 to 75 years. Other radiologic modalities are not routinely endorsed, particularly among high-risk populations, for whom colonoscopy remains the recommended standard.  Diagnostic Accuracy of CT Colonography (without IV contrast):  • American College of Radiology (ACRIN) trial: Sensitivity of 90% and specificity of 86% for polyps ≥10 mm.  • Multidetector-row CT study: Sensitivity was 91% with specificity of 93% for polyps ≥6 mm, increasing to sensitivity of 92% and specificity of 98% for polyps ≥10 mm.  • Prospective analysis: Sensitivity and specificity for detecting adenomas ≥10 mm were 91% and 85%, respectively; accuracy declined for smaller adenomas (≥8 mm: sensitivity 70%, specificity 86%; ≥6 mm: sensitivity 59%, specificity 88%).  • Meta-analyses: Report pooled sensitivities ranging from 85% to 93%, with specificity consistently around 97% for polyps ≥10 mm.  • Contrast use comparisons: Oral contrast-enhanced CTC shows higher sensitivity (78%) compared to intravenous contrast-enhanced CTC (63%), with comparable specificities of 86% and 89%, respectively.  • Prospective evaluations: Demonstrated sensitivities of 91%, 70%, and 59% for polyps ≥10 mm, ≥8 mm, and ≥6 mm respectively, with corresponding specificities ranging between 85% to 88%. |
| Colorectal Cancer Screening: Recommendations for Physicians and Patients from the U.S. Multi-Society Task Force on Colorectal Cancer – 2017 ^14^, United States | 50 years for most average-risk persons; 45 years for African Americans | 75 years for those up to date with screening and with prior negative results; up to 85 years for those without prior screening, depending on age and comorbidities | - Colonoscopy: every 10 years  - FIT: annually  - CT colonography: every 5 years  - Flexible sigmoidoscopy: every 5 to 10 years  - FIT-fecal DNA: every 3 years  - Capsule colonoscopy: every 5 years (third-tier, limited use) | The paper updates colorectal cancer screening recommendations by ranking tests into three tiers, emphasizing colonoscopy every 10 years and annual FIT as primary options, and provides guidelines on starting and stopping screening based on age, risk factors, and family history to reduce CRC incidence and mortality.  1. Colonoscopy every 10 years  2. Annual fecal immunochemical test (FIT)  3. CT colonography every 5 years  4. FIT-fecal DNA every 3 years  5. Flexible sigmoidoscopy every 5 to 10 years  6. Capsule colonoscopy every 5 years (if available)  - Reduction in CRC incidence and mortality with colonoscopy:    - Distal colon: ~80%    - Proximal colon: 40% to 60%  - Sensitivity and specificity of tests:    - FIT-fecal DNA test: Sensitivity for CRC: 92%, Specificity: 86.6% to 89.8%    - FIT: Sensitivity for cancer: 79%, Sensitivity for advanced adenomas: ~30%, Specificity: 96%    - Septin9 assay: Sensitivity for CRC: 48%, No sensitivity for precancerous polyps  - Risks associated with colonoscopy:    - Perforation: 0.5 per 1000    - Bleeding: 2.6 per 1000    - Death: 2.9 per 100,000. |
| AGA Clinical Practice Update on Risk Stratification for Colorectal Cancer Screening and Post-Polypectomy Surveillance: Expert Review – 2023 ^15^, United States, Sweden, Nordic-European (multinational) | Colorectal cancer screening should commence at 45 years of age for average-risk adults; however, individuals with a positive family history should initiate screening either at age 40 or ten years prior to the age at diagnosis of the youngest affected relative, whichever occurs first. | The decision to discontinue CRC screening is individualized for those older than 75 years, based on risks, benefits, screening history, and comorbidities. There is no specific discontinuation age mentioned. | Recommended colorectal cancer screening intervals for average-risk individuals include:  • Colonoscopy every 10 years.  • Annual fecal immunochemical testing (FIT).  • Flexible sigmoidoscopy at intervals of 5 to 10 years.  • Multitarget stool DNA-FIT testing every 3 years.  • Computed tomography colonography (CTC) every 5 years. | The guidelines present evidence-based recommendations for colorectal cancer (CRC) screening, emphasizing the importance of individual risk stratification. Individuals identified with an increased familial risk should begin screening at age 40 or ten years earlier than the youngest family member diagnosed with CRC. For adults at average risk, screening is recommended to commence at age 45, with suggested modalities including colonoscopy, fecal immunochemical testing (FIT), flexible sigmoidoscopy combined with FIT, multitarget stool DNA-FIT testing, or computed tomography colonography (CTC).  - Relative risk (RR) of developing CRC if at least one FDR is affected:    - Meta-analysis (2001-2006): RR = 2.24 to 2.26    - Meta-analysis (2018): RR = 1.76  - RR when FDR diagnosed before age 50:    - Case-control studies: RR = 3.57    - Cohort studies: RR = 3.26  - RR when FDR diagnosed after age 50:    - Case-control studies: RR = 1.88    - Cohort studies: RR = 1.83  - RR for individuals with an FDR with any adenomatous polyp: RR = 4.36  - Odds ratio for individuals with an FDR with adenomas ≥1 cm: OR = 2.27  - RR for FDRs of individuals with any adenomas: RR = 1.78  - Odds ratio for having an FDR with any type of colorectal polyp: OR = 1.40  - Prevalence of advanced adenomas among siblings of patients with advanced adenomas: OR = 6.05  - Cost-effectiveness of starting screening at age 45:    - Colonoscopy: $33,900 per quality-adjusted life-year gained    - FIT: $7,700 per quality-adjusted life-year gained  - Hazard ratio for lower endoscopy initiation:    - Before age 45: HR = 0.37    - Age 45-49: HR = 0.43 |
| Early Detection for Colorectal Cancer: ASCO Resource-Stratified Guideline – 2019 ^16^, global (encompassing various regions such as North America, Australia, New Zealand, Western Europe, Japan, and South Korea) | 50 years | 75 years | Recommended colorectal cancer screening modalities include annual or biennial guaiac-based fecal occult blood testing (gFOBT), annual fecal immunochemical testing (FIT), flexible sigmoidoscopy every five years, and colonoscopy every ten years. Computed tomography colonography (CTC) may be considered, although optimal intervals are not explicitly defined. | Recommended Screening Modalities:  1.Guaiac-based fecal occult blood testing (gFOBT) every 1–2 years.  2.Fecal immunochemical testing (FIT) every 1 to 2 years.  3.Flexible sigmoidoscopy every 5 years.  4.Colonoscopy every 10 years as resources permit.  5.Computed tomography colonography (CTC) if colonoscopy or sigmoidoscopy is contraindicated or unavailable.  6.Endoscopic evaluation for positive stool-based screening tests.  7.Double-contrast barium enema as an alternative diagnostic modality in limited-resource settings.  8.Double-contrast barium enema as a diagnostic alternative when endoscopy is not accessible.  9.Guaiac-based fecal occult blood testing (gFOBT) every 1–2 years, achieving mortality reduction rates of approximately 22% when conducted biennially, increasing to 32% with annual screening.  10.Fecal immunochemical testing (FIT) every 1-2 years, providing sensitivity and specificity comparable to guaiac-based testing, but associated with higher patient adherence rates.  11Multitarget stool DNA (FIT-DNA) testing is conditionally recommended, given lower specificity compared to FIT. |
| Updates on Age to Start and Stop Colorectal Cancer Screening: Recommendations From the U.S. Multi-Society Task Force on Colorectal Cancer – 2022 ^17^, United States | 45 years | 75 years  76-85 – individualized |  | The paper discusses guidelines and recommendations for colorectal cancer screening in average-risk individuals |
| World Gastroenterology Organisation/International Digestive Cancer Alliance  Practice Guidelines:  Colorectal cancer screening – 2007 ^18^, global (encompassing regions such as North America, Australia, New Zealand, select European countries, and Japan) | 50 years |  | Colorectal cancer screening recommendations include annual testing with either guaiac-based fecal occult blood tests (gFOBT) or fecal immunochemical tests (FIT). Additionally, endoscopic methods, such as flexible sigmoidoscopy every five years or colonoscopy every ten years, are advised as standard options. | Recommended Colorectal Cancer Screening Modalities:  1.Colonoscopy, every 10 years, starting at age 50 for average-risk adults.  2.Flexible sigmoidoscopy, at regular intervals as appropriate.  3.Fecal occult blood testing (FOBT), employing either guaiac-based methods or fecal immunochemical tests (FIT).  4.Fecal DNA testing, recommended as an alternative method for early CRC detection.  5.Computed tomography colonography (CTC), suggested as a viable screening alternative where appropriate.  - Fecal occult blood test:    - Mortality reduction: 15-33% in cohorts, 45% in compliers    - Sensitivity: 50-60% one-time, up to 90% with regular use  - DNA panel test:    - Sensitivity: 52%    - Specificity: 94.4%  - Guaiac smear test:    - Sensitivity: 13%    - Specificity: 95.2%  - Sigmoidoscopy:    - Mortality reduction: 60-70%  - Postpolypectomy follow-up:    - Incidence and mortality reduction: 90%  - Computed-tomographic colonography (CTC):    - Sensitivity: 93% for large polyps    - Specificity: 97% |
| Screening for Colorectal Cancer: US Preventive Services Task Force Recommendation Statement US – 2021 ^19^, United States | 45 years | 75 years  > 76 individualized | - gFOBT: every year  - FIT: every year  - sDNA-FIT: every 1 to 3 years  - CT colonography: every 5 years  - Flexible sigmoidoscopy: every 5 years or every 10 years with annual FIT  - Colonoscopy: every 10 years | The US Preventive Services Task Force recommends colorectal cancer screening for adults aged 45 to 75 years, with selective screening for those aged 76 to 85, based on individual health and preferences, reflecting updated guidelines to address increasing incidence rates.  1. High-sensitivity guaiac fecal occult blood test (HSgFOBT) every year  2. Fecal immunochemical test (FIT) every year  3. Stool DNA-FIT every 1 to 3 years  4. Computed tomography colonography every 5 years  5. Flexible sigmoidoscopy every 5 years  6. Flexible sigmoidoscopy every 10 years + annual FIT  7. Colonoscopy screening every 10 years  Impact and Adverse Outcomes Associated with Colorectal Cancer Screening (ages 45–75 years):  •Life-years gained: approximately 286 to 337 per 1,000 adults screened.  •CRC cases prevented: approximately 42 to 61 per 1,000 adults screened.  •CRC deaths prevented: approximately 24 to 28 per 1,000 adults screened.  •Impact of initiating screening at age 45 (versus 50): increases life-years gained by 22 to 27 per 1,000 adults screened, equivalent to 8 to 10 additional days per individual.  •Complications of colonoscopy:  •Serious bleeding events: approximately 17.5 per 10,000 procedures (95% CI, 7.6–27.5).  •Colonic perforation: approximately 5.4 per 10,000 procedures (95% CI, 3.4–7.4). |
| Colorectal cancer screening with faecal immunochemical testing, sigmoidoscopy or colonoscopy: a clinical practice guideline – 2019 ^20^ | 50 years | 79 years | Recommended colorectal cancer screening approaches include:  •Annual or biennial fecal immunochemical testing (FIT).  •One-time flexible sigmoidoscopy.  •One-time colonoscopy. | The study evaluates colorectal cancer screening methods for adults aged 50–79 years, affirming that recent evidence supports existing guidelines without necessitating substantial changes. It emphasizes individualized screening decisions based on personal risk assessment. Recommended screening approaches include annual or biennial fecal immunochemical testing (FIT), or a single examination with flexible sigmoidoscopy or colonoscopy. |

**Appendix B - Questionnaires for the Round I and Round II: SCREECO project: Colorectal cancer SCREEning implementation pathways across developed versus developing healthcare systems: a modified Delphi COnsensus**

**Appendix B.1**

Questionnaire for the Round I: SCREECO project: Colorectal cancer SCREEning implementation pathways across developed versus developing healthcare systems: a modified Delphi COnsensus

SCREECO project: Colorectal cancer SCREEning implementation pathways across developed versus developing healthcare systems: a modified Delphi Consensus
Over the past 30 years, the incidence and mortality of colorectal cancer have doubled globally, with a significant increase in younger adults (less than 50 years).
75% of colorectal cancer disability-adjusted life years (DALYs) are distributed in Low and middle Socio-Demographic Index countries, and further increases are forecasted.
Colorectal cancer screening is associated with a reduction in mortality from this disease. However, the development of a screening program at the national level has multiple human and material limitations, which lead to a reduced compliance / adherence / implementation rate.
Please fill in the form below, whose analysis will present the perception of this approach in different groups of population, both professionals and patients (Round 1 of voting for this questionnaire). In Round 2, questions for which the consensus exceeds 70% will be resubmitted, along with other questions proposed by you / the core group of specialists.
Through your clinical experience you can significantly contribute to the proposal of a nationally standardized protocol, with a degree of applicability and increased impact on the general population.

The form that we invite you to fill in is addressed to all health professionals (doctors, resident doctors, students), patients and healthy people from the general population.
Your participation in the study is voluntary and you may withdraw from the study at any time.
The duration of the questionnaire is approximately 10 minutes.
 If you have questions about this study, you can email them to [ionut.negoi@umfcd.ro](mailto:ionut.negoi@umfcd.ro).
By completing this questionnaire, you agree to voluntarily participate in this study and confirm that you have read and understood all of the above information. If you would like to receive the Round 2 questionnaire as well, please complete your e-mail. All those who participate in both rounds will be acknowledged in the published materials resulting from this analysis.

   Thank you for completing this questionnaire!

E-mail*

Country*

Surname

First name

Affiliation

Age *

Specialty *

Years of clinical experience

Hospital type for practice*

Q1: Does your country have in place a functional national screening programe for CRC?*

Yes

No

Regional not national

Q2: If YES, how effective / functional do you consider it (from one to ten)?

Q3: General Practitioner should include at the patients first visit an assessment if the patient is at increased risk for colorectal cancer*

Check all that apply (Marcați toate care se aplică)

Q 2: General Practitioner should evaluate the risks to develop a CCR starting from the patients age of:*

Check all that apply (Marcați toate care se aplică)

Q 3: General practitioner should reassess the risk of patients to develop a CCR at an interval of:*

Q 4: General Practitioner Visit assessment if the patients is at increased risk for colorectal cancer should include as standard:*

Check all that apply (Marcați toate care se aplică)

Familial history of cancer or adenomatous polyps

Personal history of CRC or benign colorectal pathology such as adenomatous polyps

Familial history of genetic syndromes associated with CRC

Personal history of ulcerative colitis or Crohn disease

Personal history of abdominal radiation for pediatric cancers

Q 5: Patients with no risk factors from the above question fall in the average risk population *

Check all that apply

Agree

Disagree

Q 6: Suggested risk factors to be included in the general practitioner evaluation

Check all that apply

Q 7: Age for starting screening in average risk population *

Q 8: Do you recommend as frequency for the screening in AVERAGE risk population:*

Q 9: Age for starting screening in high risk population *

Q 10: Do you recommend as frequency for the screening in HIGH risk population:*

Q 11: Screening discontinuation should be at the age of:

Q 12: Screening methods which maybe considered for a national implementation*

Fecal immunochemical tests (FIT)

Guaiac-based fecal occult tests

Colonoscopy

Sigmoidoscopy

Computed Tomography colonography

Q 13: Estimated sensitivity for colorectal cancer of the following screening methods is:

70%-75%

75%-80%

80%-85%

85%-90%

90%-95%

95%-100%

Fecal immunochemical tests every year (FIT)

Guaiac-based fecal occult tests every year

Colonoscopy every 10 years

Sigmoidoscopy every 5 years

Computed Tomography colonography

Fecal immunochemical tests every year (FIT)

Guaiac-based fecal occult tests every year

Colonoscopy every 10 years

Sigmoidoscopy every 5 years

Computed Tomography colonography

Q 14: Estimated prevented death due to colorectal cancer per 1000 people older than 40 years

10-15

15-20

20-25

25-30

Fecal immunochemical tests every year (FIT)

Guaiac-based fecal occult tests every year

Colonoscopy every 10 years

Sigmoidoscopy every 5 years

Computed Tomography colonography every 5 years

Fecal immunochemical tests every year (FIT)

Guaiac-based fecal occult tests every year

Colonoscopy every 10 years

Sigmoidoscopy every 5 years

Computed Tomography colonography every 5 years

Q 15: What screening strategy will you choose for you as a patient:*

Q 16: What screening strategy will you consider with a maximum of adherence for patients from urban areas:

Q 17: What screening strategy will you consider with a maximum of adherence for patients from rural areas:

Q 18: What screening strategy will you choose in case of limited acces to colonoscopy:

Q 19: In high risk patients what screening strategy will you choose for you as a patient:

Every year (Anual)

Every two year (La fiecare 2 ani)

Every three years (La fiecare 3 ani)

Colonoscopy

Fecal immunochemical tests / CT colonography THAN colonoscopy in the case of a positive initial test

Others (sigmoidoscopy with fecal immunochemical tests OR sigmoidoscopy alone OR capsule colonoscopy)

Colonoscopy

Fecal immunochemical tests / CT colonography THAN colonoscopy in the case of a positive initial test

Others (sigmoidoscopy with fecal immunochemical tests OR sigmoidoscopy alone OR capsule colonoscopy)

Q 20: What costs do you think are for a colonoscopy (all hidden costs, including single use medical devices, human resources, service and technologies depreciation, etc.)?*

Q 21: Do you consider public sector reimbursement for colonoscopy to cover all the costs? *

Q 22: Do you consider that reimbursement from public sector of all the costs for screening colonoscopy and early treatment will decrease the costs for healthcare system (decreasing the costs for medical care for patients with advanced disease, decreasing the burden of the disease in the society, increasing the contribution for society of treated patients, etc)?*

Q 23: Do you consider for your country to start screening directly with colonoscopy in high risk population, as defined by general practitioner?

Q 24: Do you agree that all hospitals  / available resources should be involved in screening?*

Yes

No

Q 25: What number of general populations should be allocated to a screening unit (human and material resources dedicated to screening in a hospital)?*

Q 26: Do you consider appropriate to implement a national programing software to schedule patients for screening ? *

Q 27: Do you have any suggestions / questions proposal for the second round of voting?

Appendix B.2

Questionnaire for the Round II: 2nd round SCREECO project: Colorectal cancer SCREEning implementation pathways across developed versus developing healthcare systems: a modified Delphi COnsensus

SCREECO project - 2nd ROUND

Dear friends & colleagues, thank you so much for investing your time to define the most effective pathways for colorectal cancer screening in different geographic and social scenarios.  
Please find below for the second round of voting the questions with less than 70% consensus.
For additional informations please e-mail to [ionut.negoi@umfcd.ro](mailto:ionut.negoi@umfcd.ro)

E-mail*

Country*

Surname

First name

Affiliation

Age *

Specialty *

Years of clinical experience

Hospital type for practice*

General Practitioner should evaluate the risks to develop a CCR starting from the patients age of 20-25 years old. *


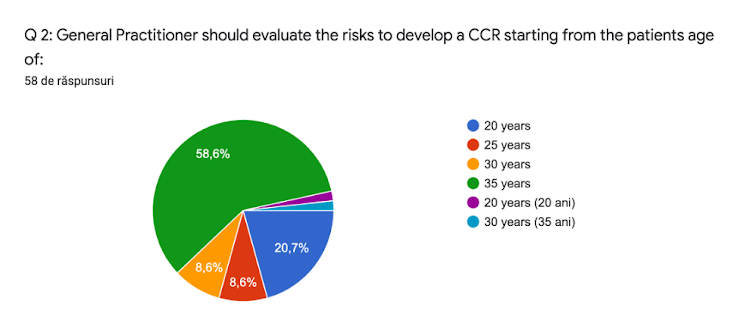


See above the results of the first voting round.

General practitioner should reassess the risk of patients to develop a CCR at an interval of 3 (three) years.*


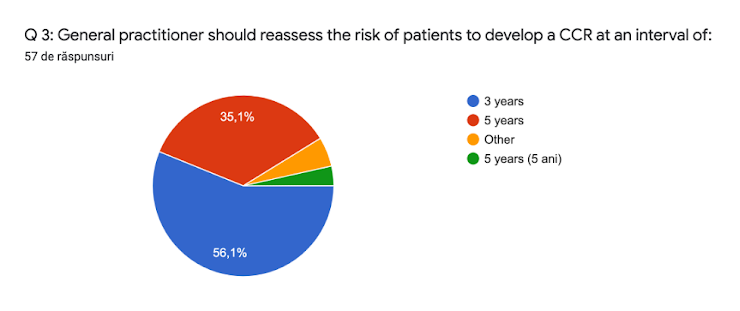


See above the results of the first voting round.

General Practitioner assessment if the patients is at increased risk for colorectal cancer should include as standard the following: (1) Familial history of cancer or adenomatous polyps; (2) Personal history of CRC or benign colorectal pathology such as adenomatous polyps; (3) Familial history of genetic syndromes associated with CRC; (4) Personal history of ulcerative colitis or Crohn disease; (5) Personal history of abdominal radiation for pediatric cancers.*


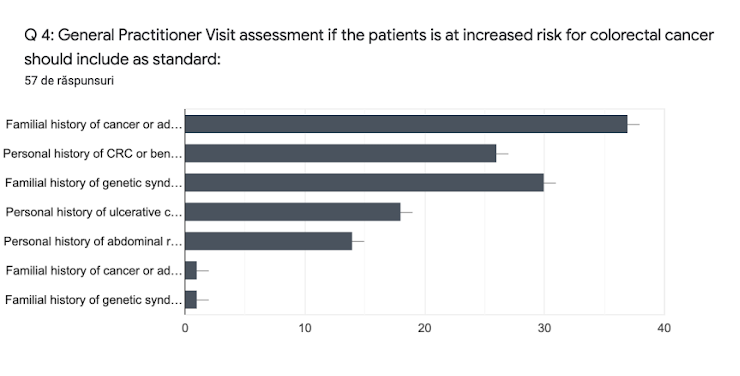


See above the results of the first voting round.

Age for starting screening in average risk population should be 45 years old. *


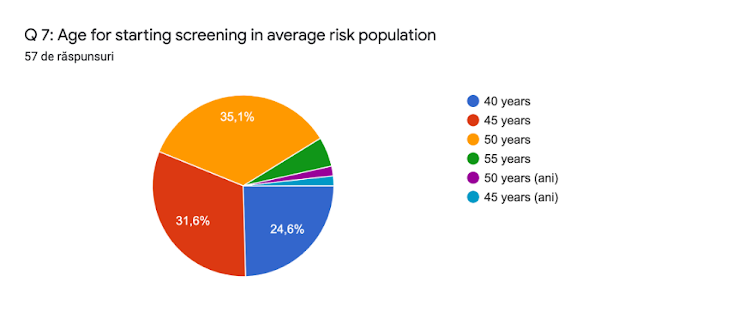


See above the results of the first voting round.

Screening discontinuation should be at the age of 75 years old. *


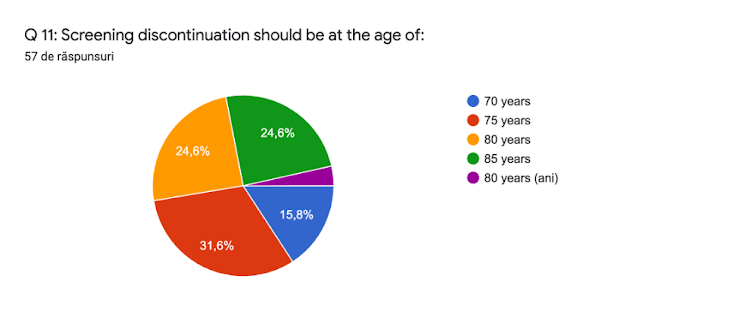


Do you agree with the below pathways, divided for urban (better- or less-informed) and rural (high- and low-population) areas? *


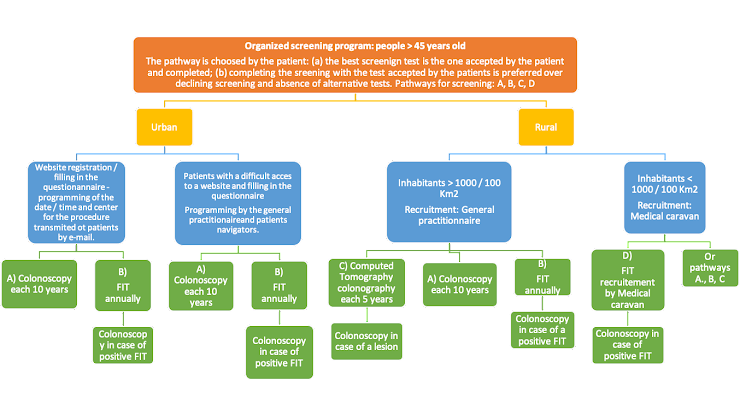


Do you have any suggestions / questions proposal for the third round of voting / final consensus meeting?

Appendix C – Figures of responses from the first and second voting rounds.

**Appendix C.1 - Figures of responses from the first voting round**


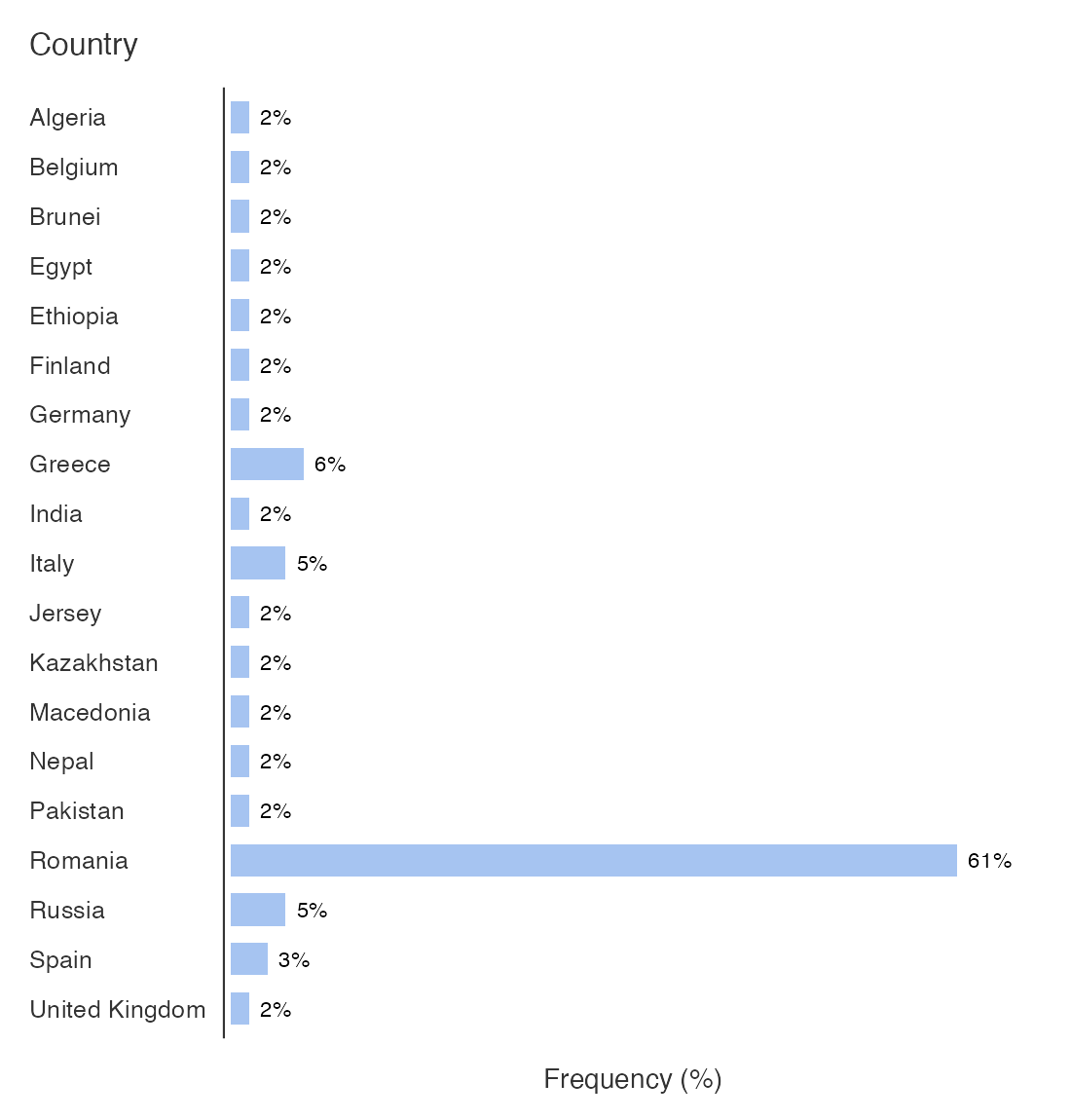


Figure C.1.1: Country of origin of participants in the Delphi consensus.


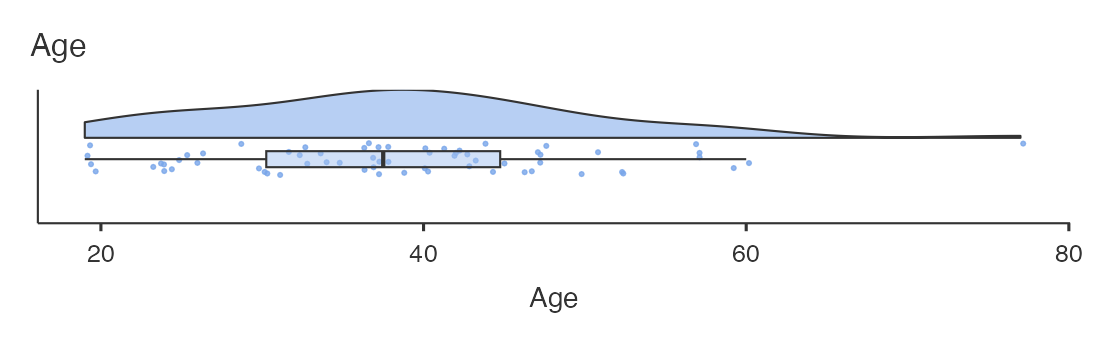


Figure C.1.2: Age of participants in the Delphi consensus.


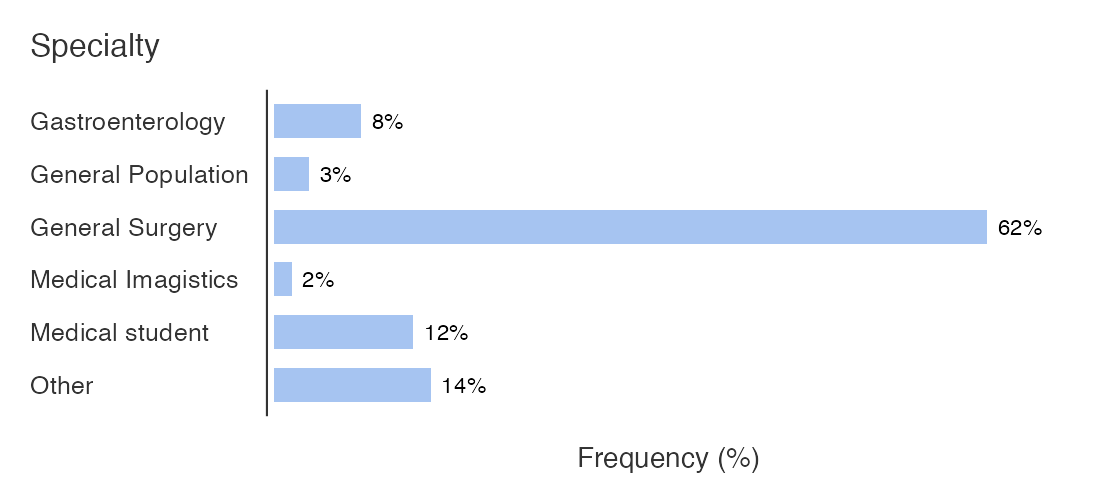


Figure C.1.3: Specialty of participants to the Delphi consensus.


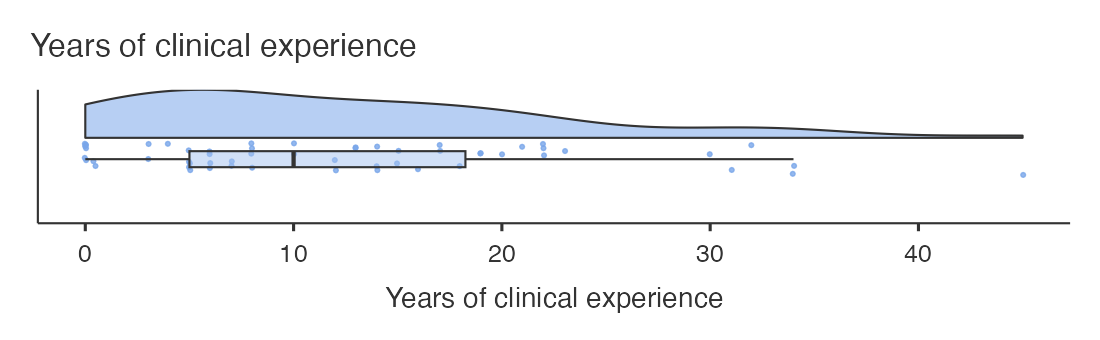


Figure C.1.4: Years of clinical experience for participants to the Delphi consensus.


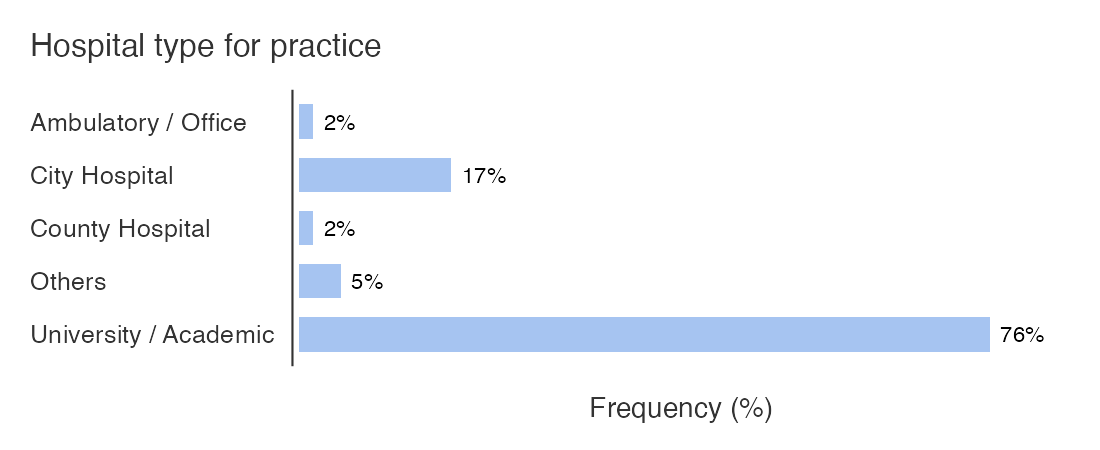


Figure C.1.5: Types of working hospitals for participants in the Delphi consensus.


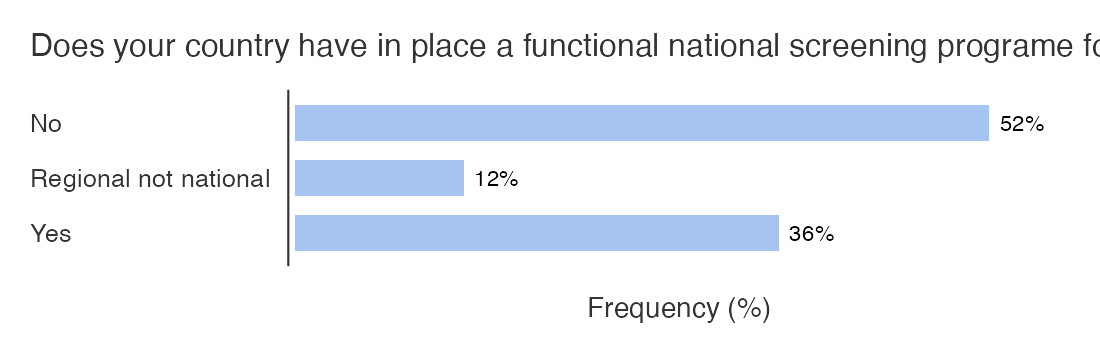


Figure C.1.6: Responses to question ”Does your country have in place a functional national screening program for CRC?”


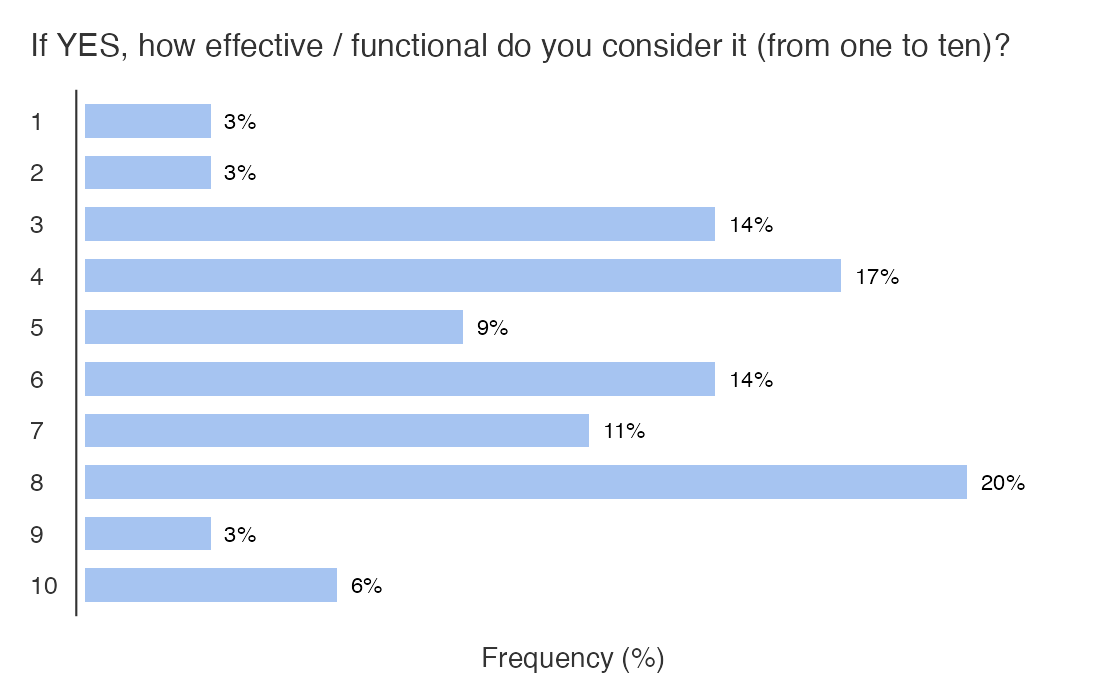


Figure C.1.7: Responses to question ” If YES, how effective / functional do you consider it (from one to ten)?”


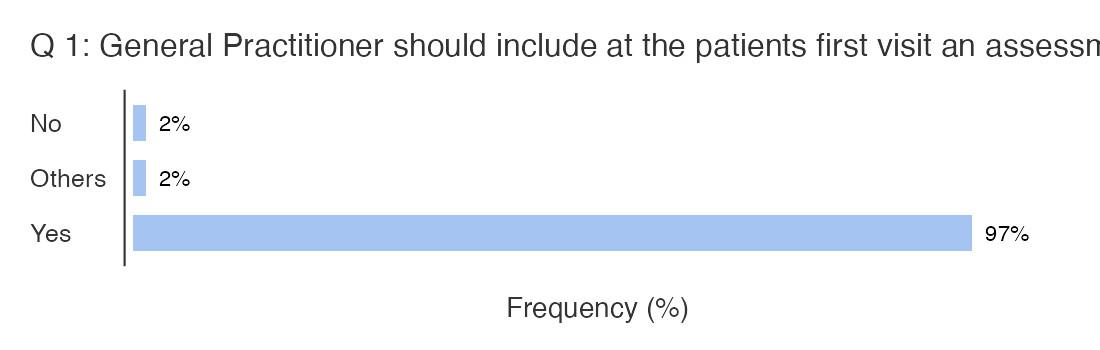


Figure C.1.8: Responses to question ” Q 1: General Practitioner should include at the patients first visit an assessment if the patient is at increased risk for colorectal cancer”


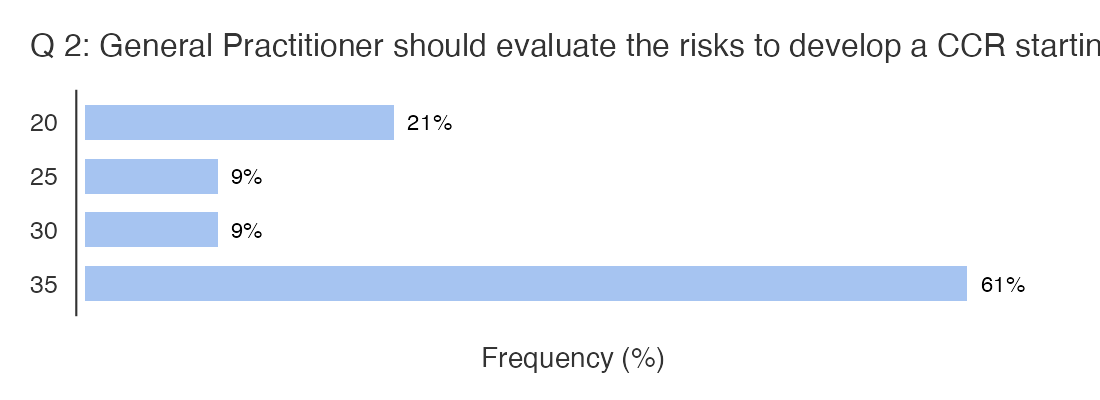


Figure C.1.9: Responses to question ” Q 2: General Practitioner should evaluate the risks to develop a CCR starting from the patients age of:”


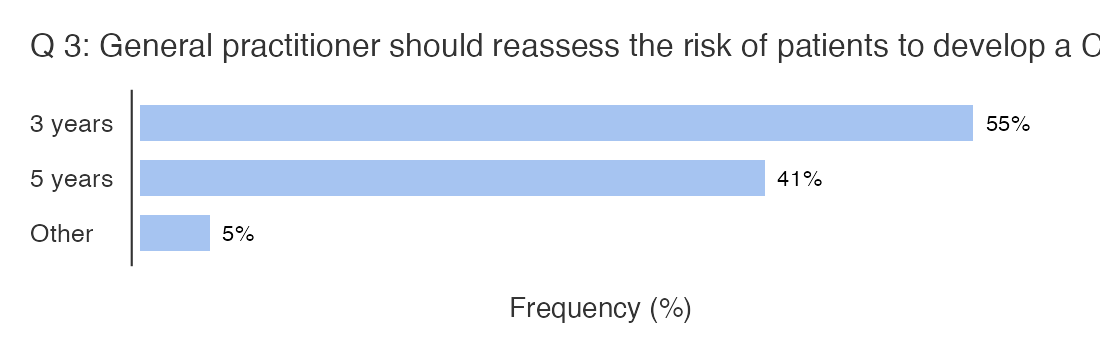


Figure C.1.10: Responses to question ” Q 3: General practitioner should reassess the risk of patients to develop a CCR at an interval of:”


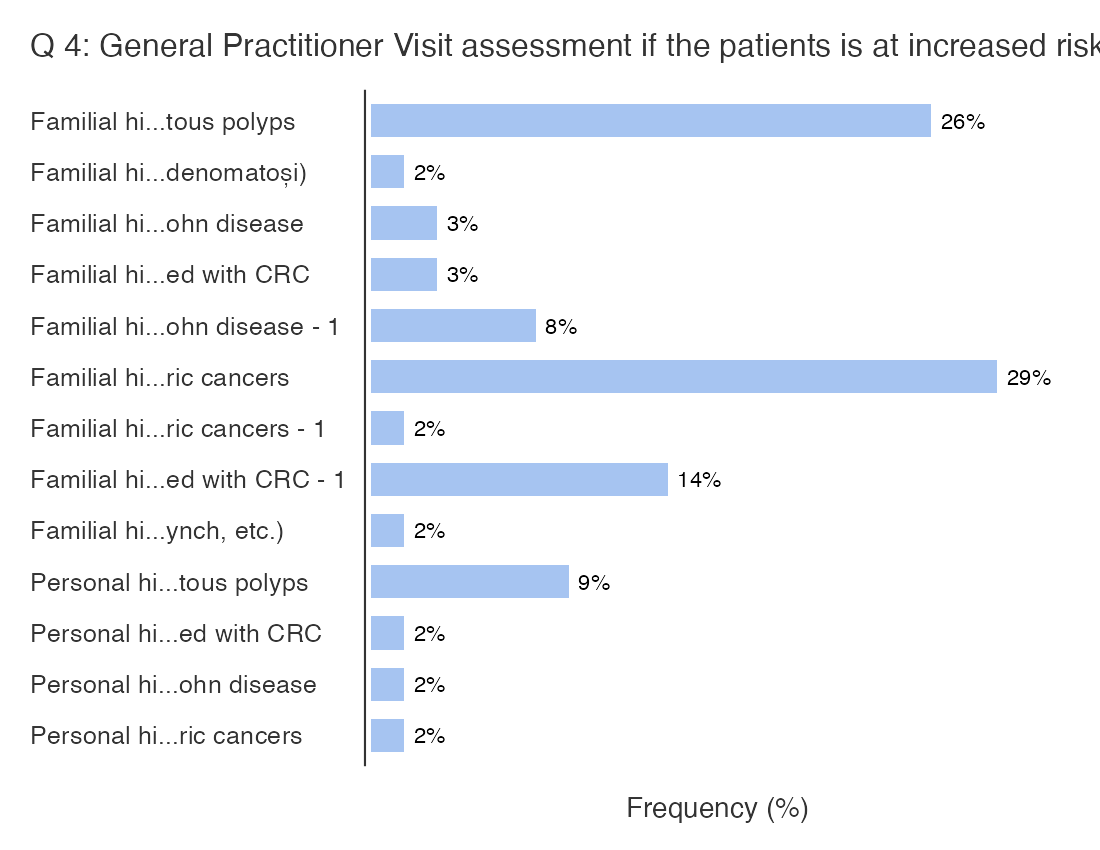


Figure C.1.11: Responses to question ” Q 4: General Practitioner Visit assessment if the patients is at increased risk for colorectal cancer should include as standard:”


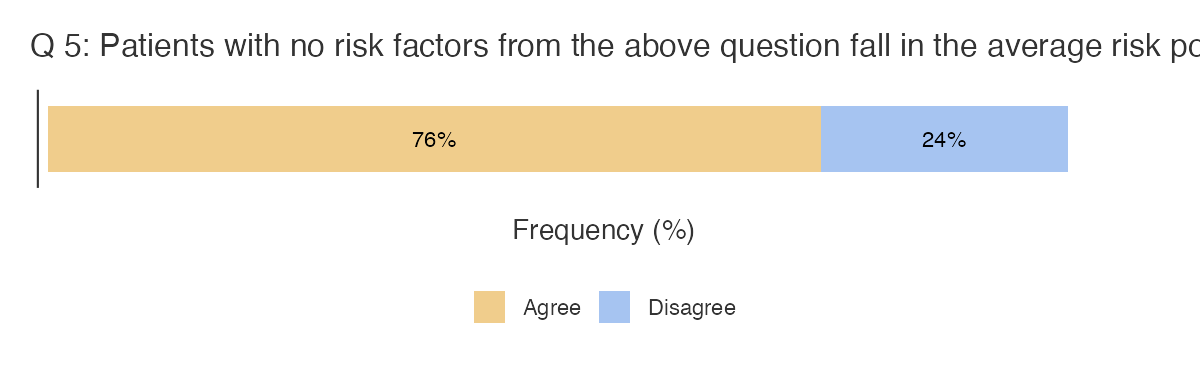


Figure C.1.12: Responses to question ” Q 5: Patients with no risk factors from the above question fall in the average risk population”

**
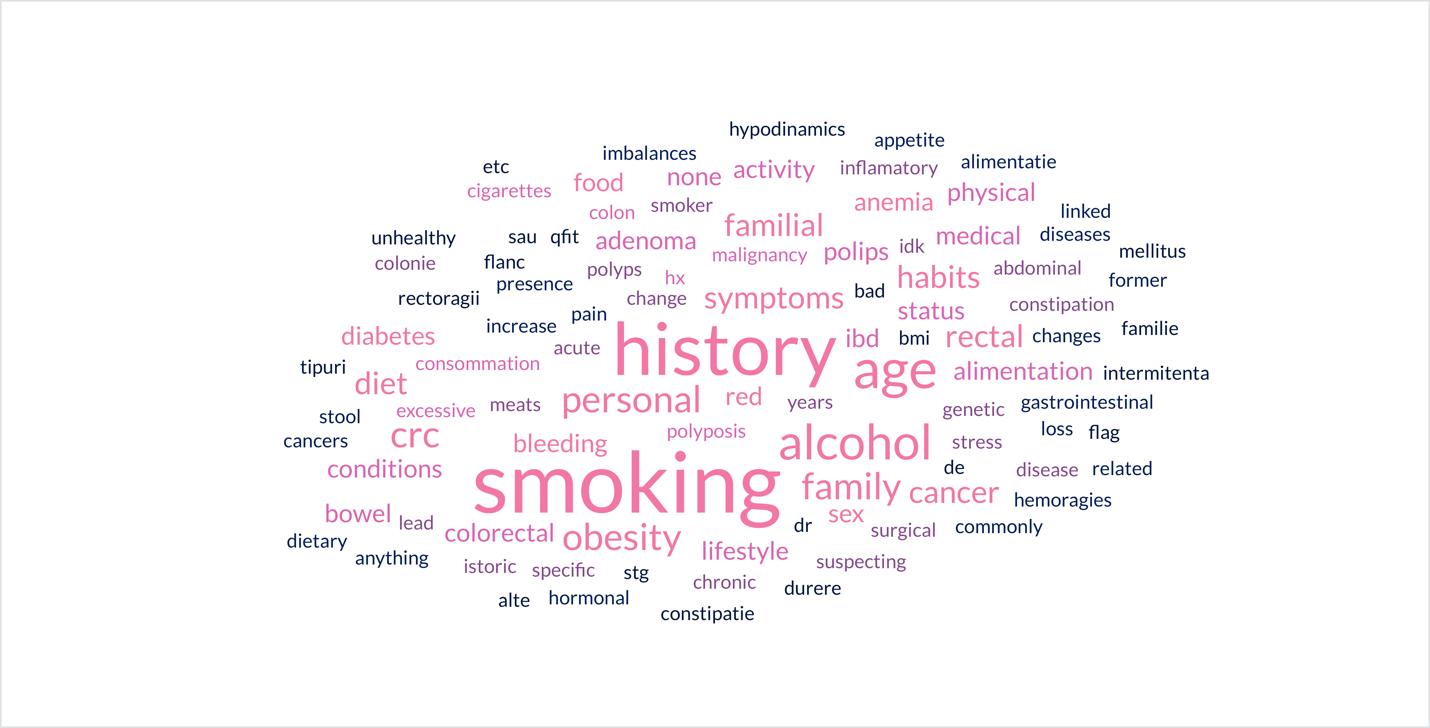
**

Figure C.1.13: Responses to question ” Q 6: Suggested risk factors to be included in the general practitioner evaluation”


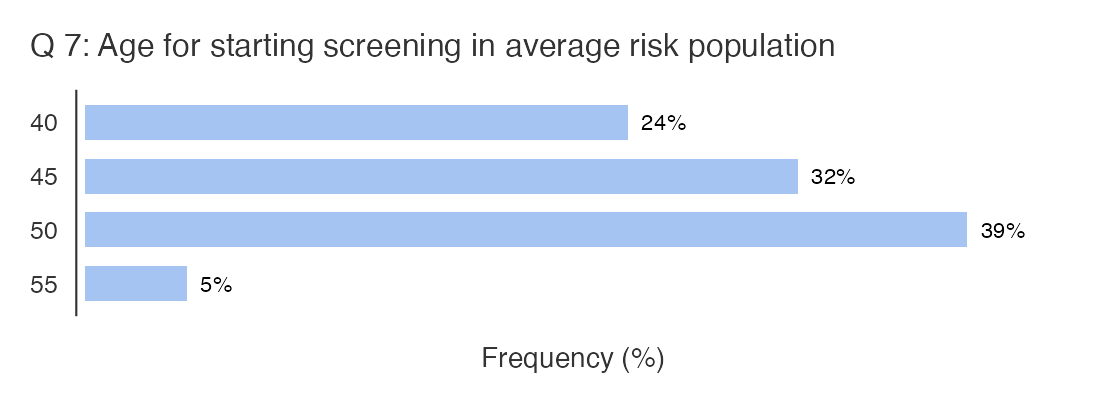


Figure C.1.14: Responses to question ” Q 7: Age for starting screening in average risk population”


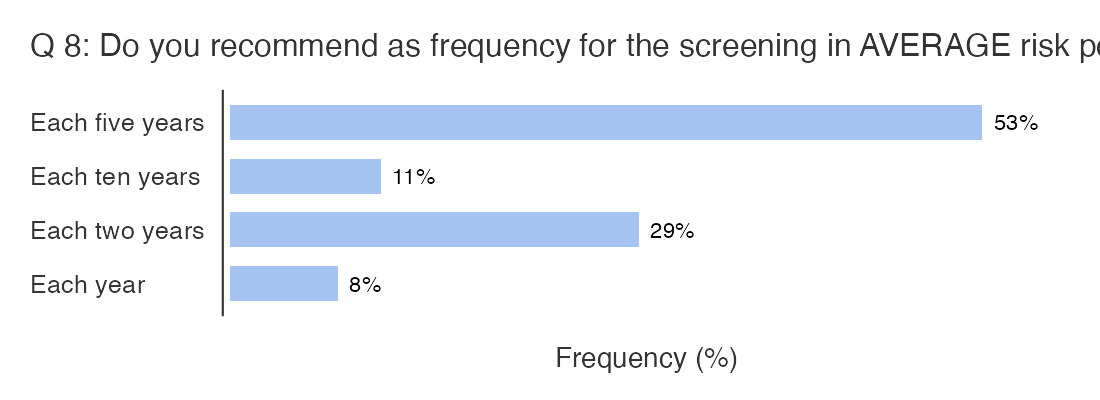


Figure C.1.15: Responses to question ” Q 8: Do you recommend as frequency for the screening in AVERAGE risk population:”


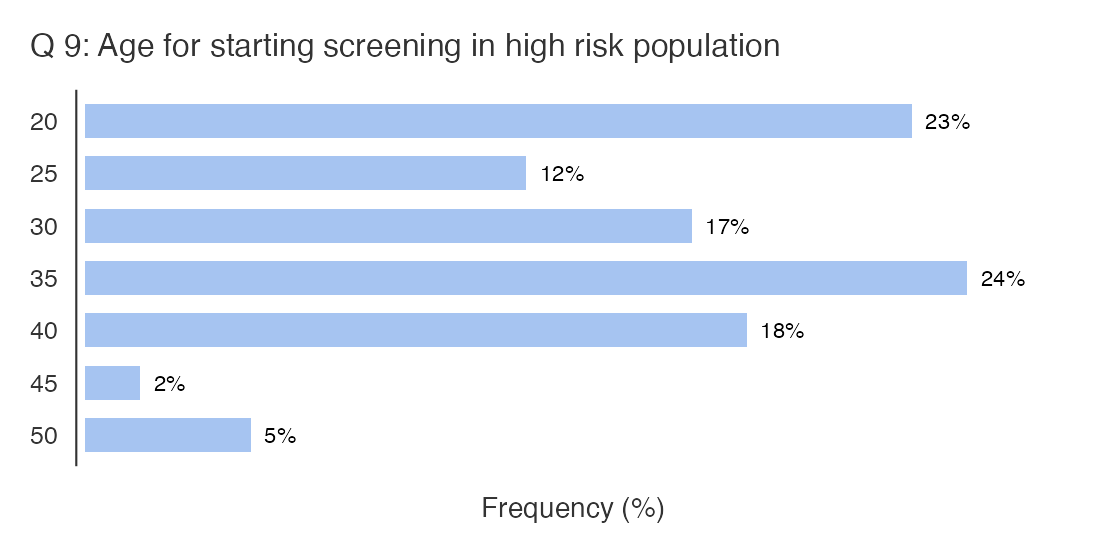


Figure C.1.16: Responses to question ” Q 9: Age for starting screening in high risk population”


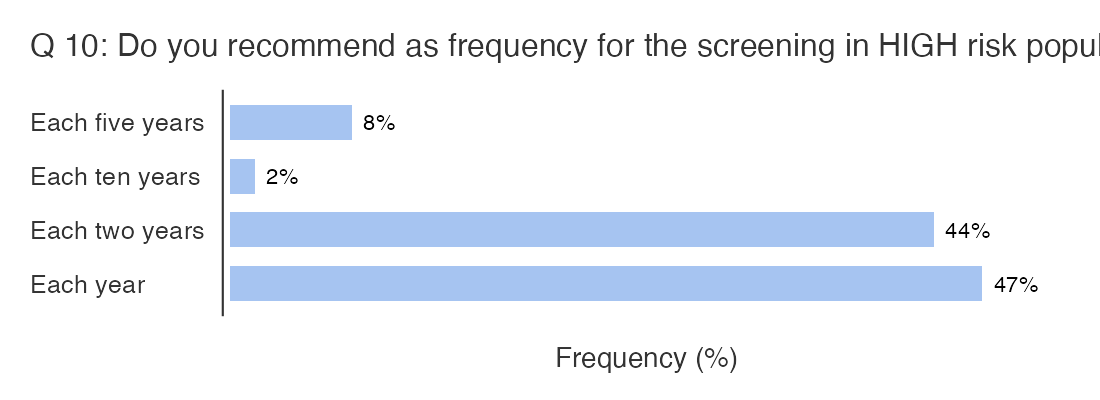


Figure C.1.17: Responses to question ” Q 10: Do you recommend as frequency for the screening in HIGH risk population:”


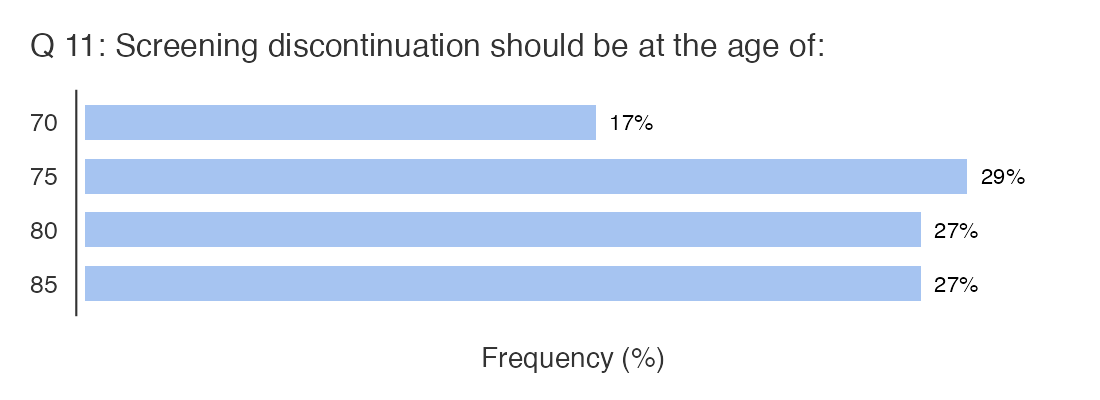


Figure C.1.18: Responses to question ” Q 11: Screening discontinuation should be at the age of:”


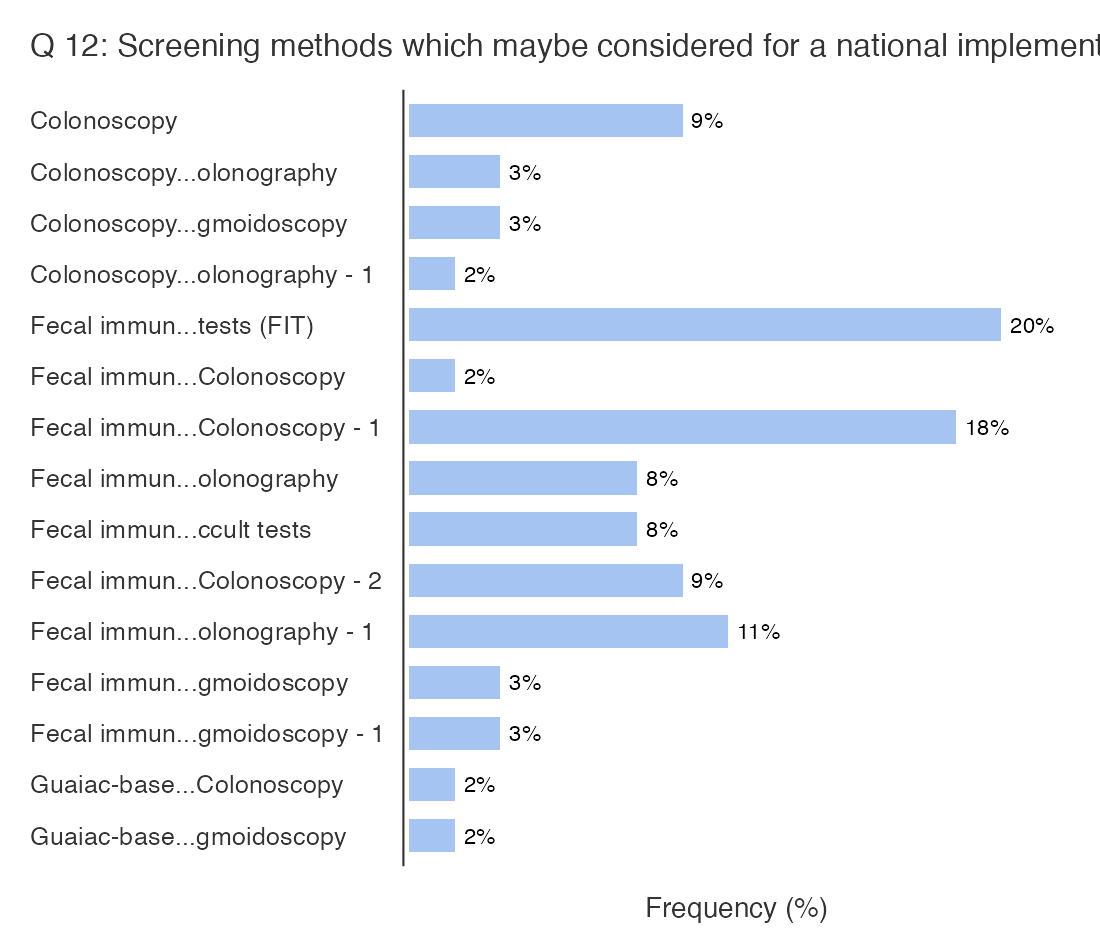


Figure C.1.19: Responses to question ” Q 12: Screening methods which may be considered for a national implementation”


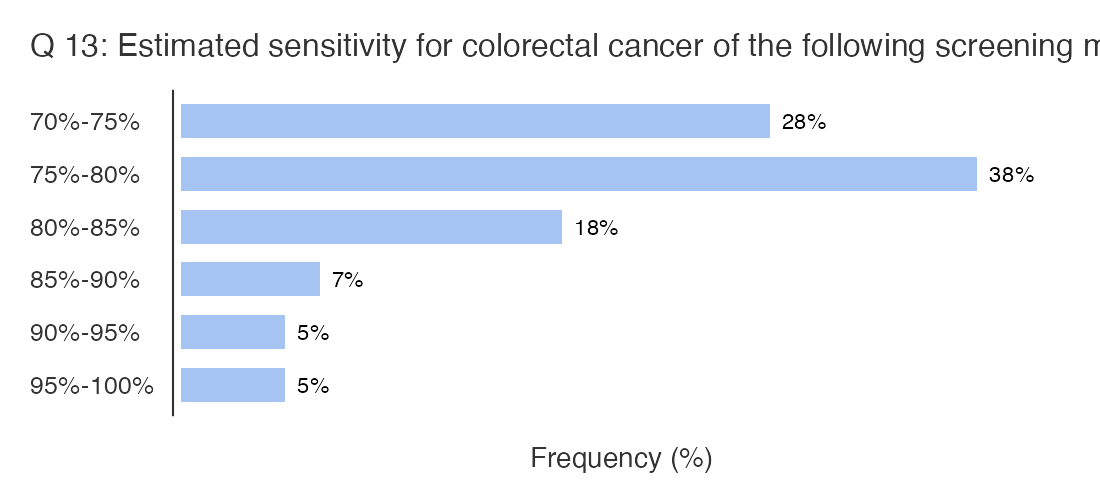


Figure C.1.20: Responses to question ” Q 13: Estimated sensitivity for colorectal cancer of the following screening methods is: [Fecal immunochemical tests every year (FIT)]”


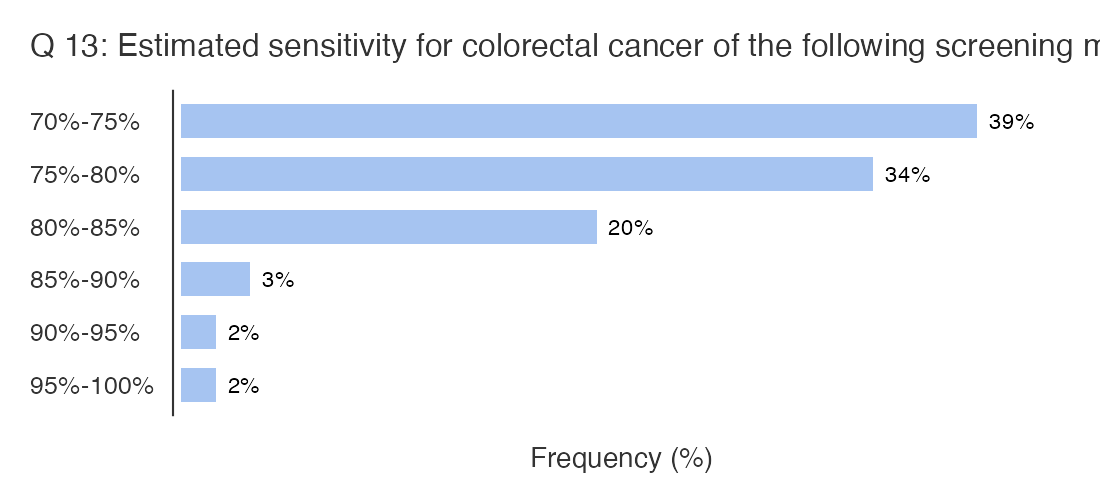


Figure C.1.21: Responses to question ” Q 13: Estimated sensitivity for colorectal cancer of the following screening methods is: [Guaiac-based fecal occult tests every year]”


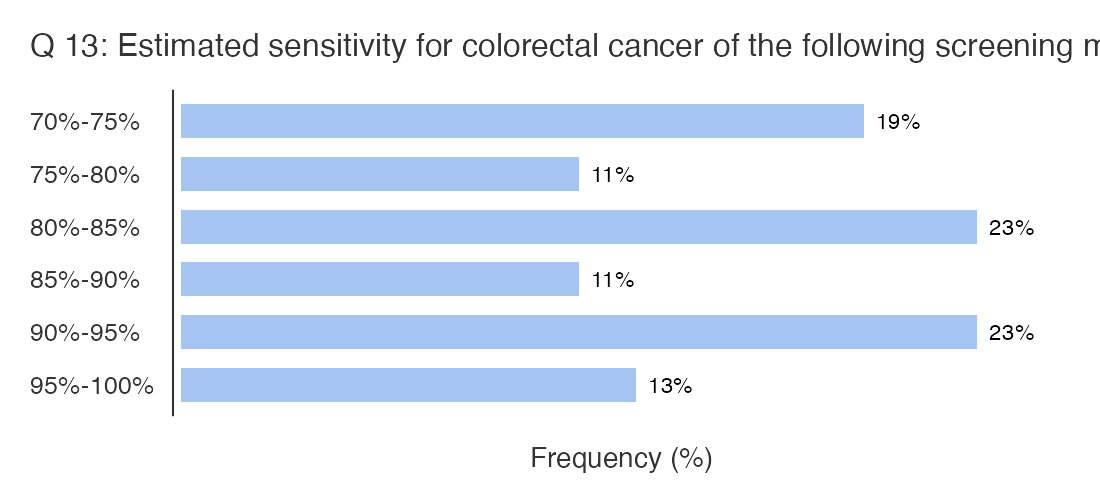


Figure C.1.22: Responses to question ” Q 13: Estimated sensitivity for colorectal cancer of the following screening methods is: [Colonoscopy every 10 years]”


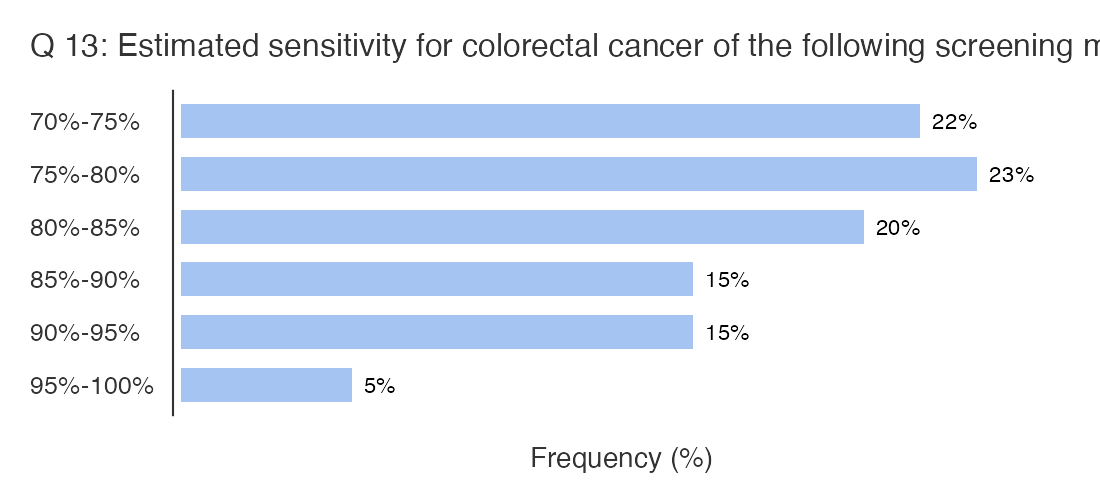


Figure C.1.23: Responses to question ” Q 13: Estimated sensitivity for colorectal cancer of the following screening methods is: [Sigmoidoscopy every 5 years]”


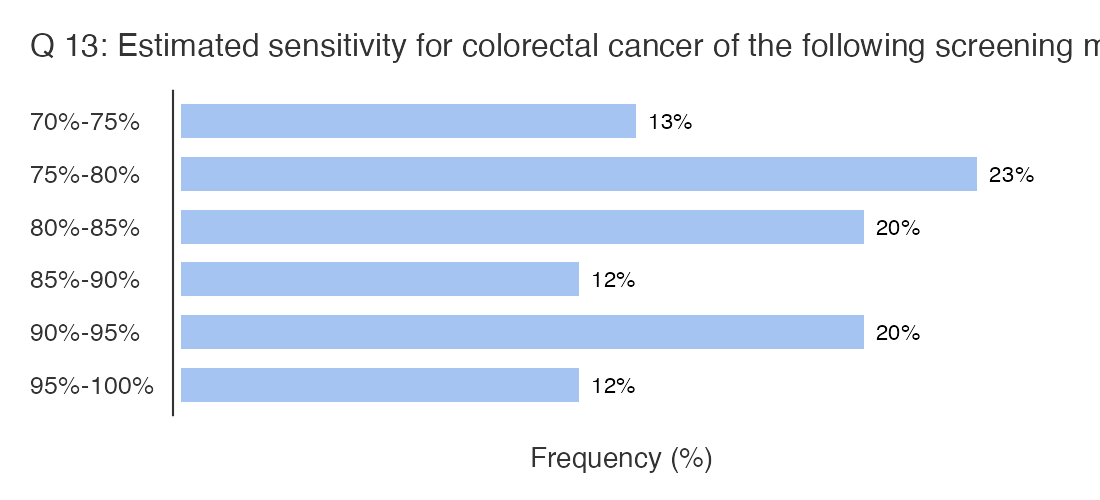


Figure C.1.24: Responses to question ” Q 13: Estimated sensitivity for colorectal cancer of the following screening methods is: [Computed Tomography colonography ]”


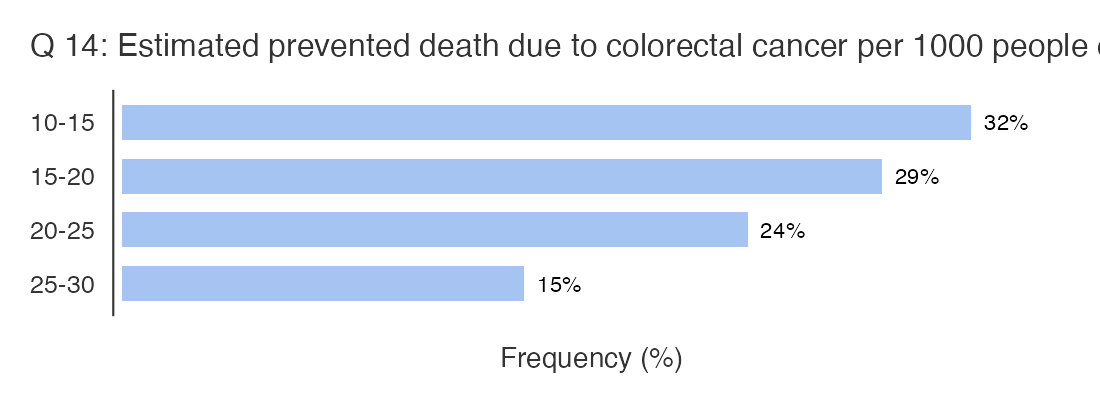


Figure C.1.25: Responses to question ” Q 14: Estimated prevented death due to colorectal cancer per 1000 people older than 40 years [Fecal immunochemical tests every year (FIT)]”


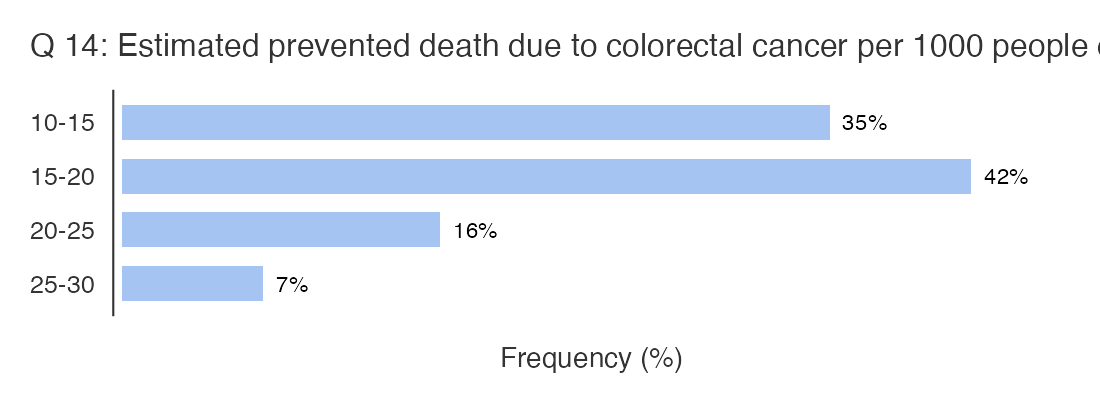


Figure C.1.26: Responses to question ” Q 14: Estimated prevented death due to colorectal cancer per 1000 people older than 40 years [Guaiac-based fecal occult tests every year]”


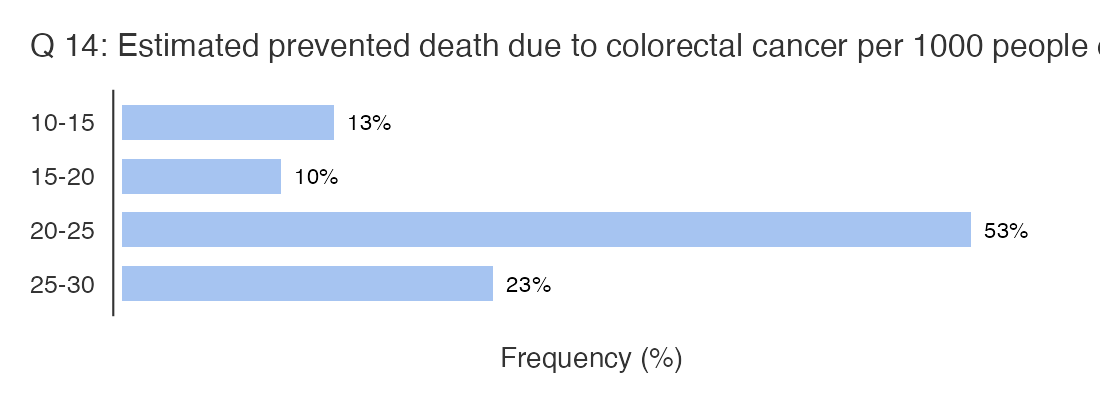


Figure C.1.27: Responses to question ” Q 14: Estimated prevented death due to colorectal cancer per 1000 people older than 40 years [Colonoscopy every 10 years]”


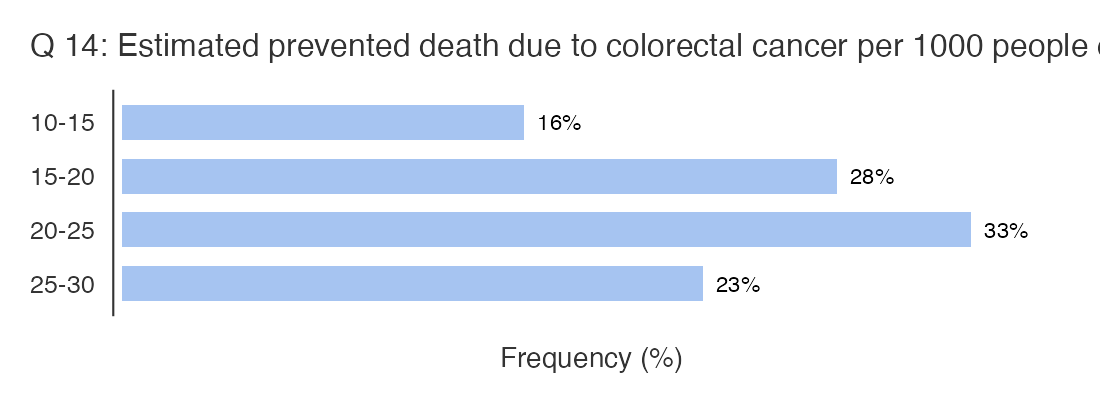


Figure C.1.28: Responses to question ” Q 14: Estimated prevented death due to colorectal cancer per 1000 people older than 40 years [Sigmoidoscopy every 5 years]”


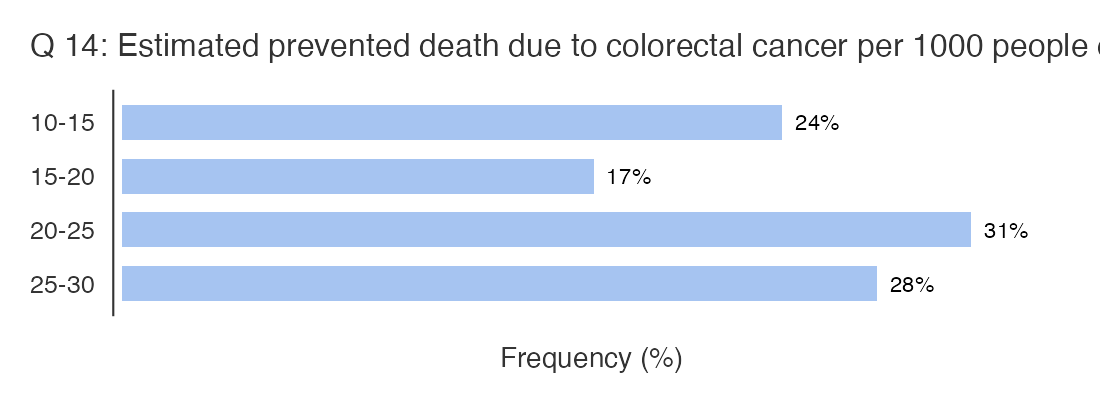


Figure C.1.29: Responses to question ” Q 14: Estimated prevented death due to colorectal cancer per 1000 people older than 40 years [Computed Tomography colonography every 5 years]”


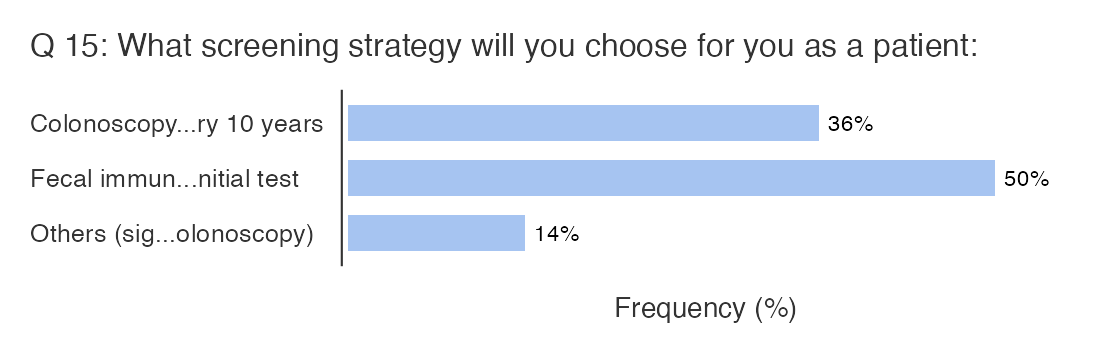


Figure C.1.30: Responses to question ” Q 15: What screening strategy will you choose for you as a patient:”


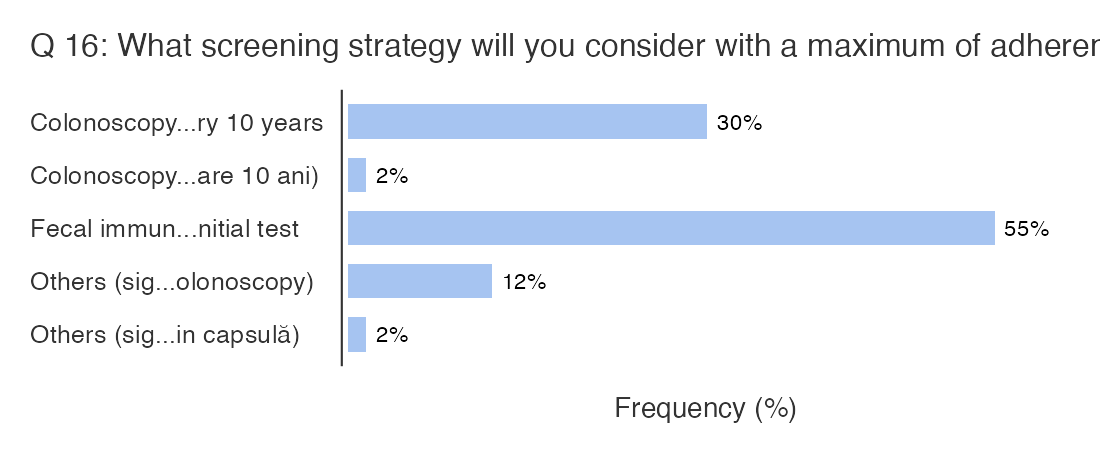


Figure C.1.31: Responses to question ” Q 16: What screening strategy will you consider with a maximum of adherence for patients from urban areas:”


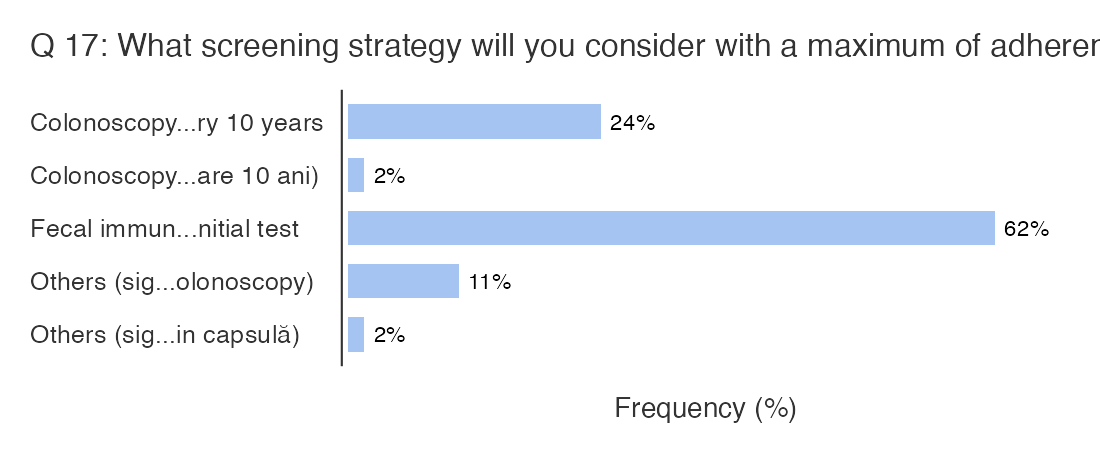


Figure C.1.32: Responses to question ” Q 17: What screening strategy will you consider with a maximum of adherence for patients from rural areas:”


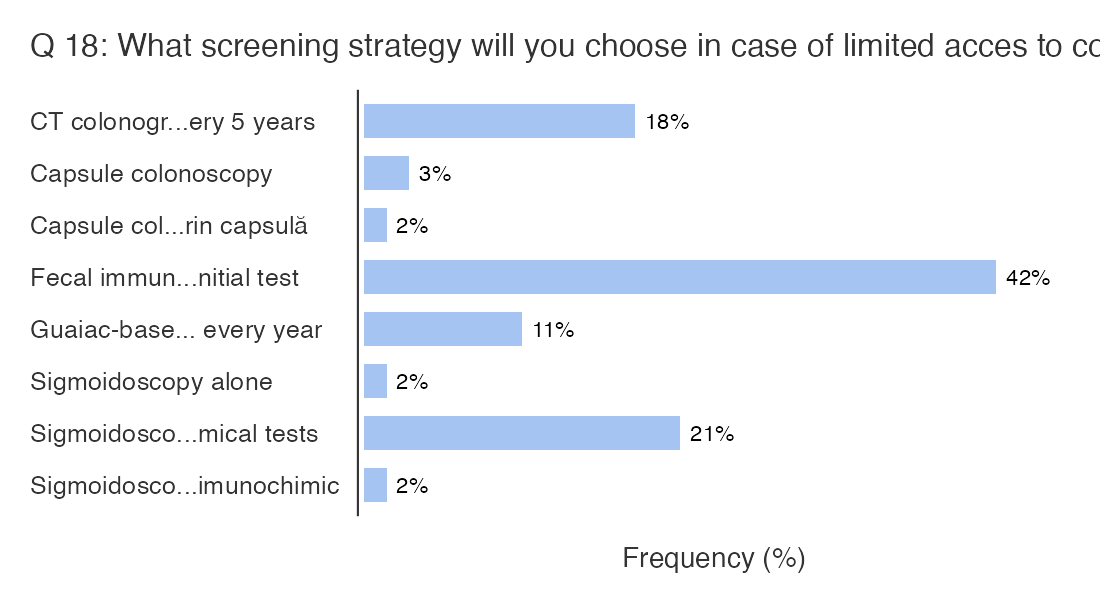


Figure C.1.33: Responses to question ” Q 18: What screening strategy will you choose in case of limited access to colonoscopy:”


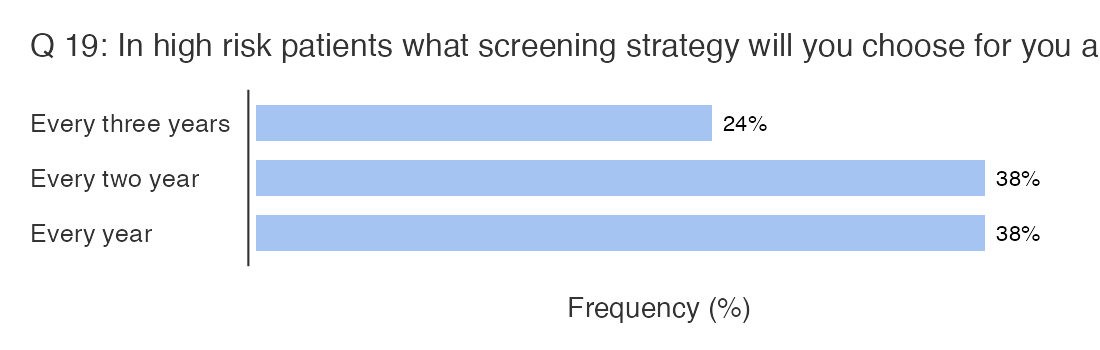


Figure C.1.34: Responses to question ” Q 19: In high risk patients what screening strategy will you choose for you as a patient: [Colonoscopy]”


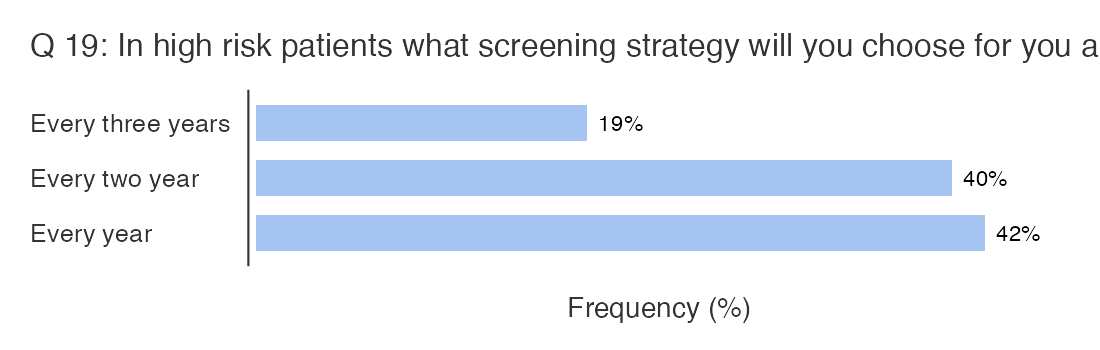


Figure C.1.35: Responses to question ” Q 19: In high risk patients what screening strategy will you choose for you as a patient: [Fecal immunochemical tests / CT colonography THEN colonoscopy in the case of a positive initial test]”


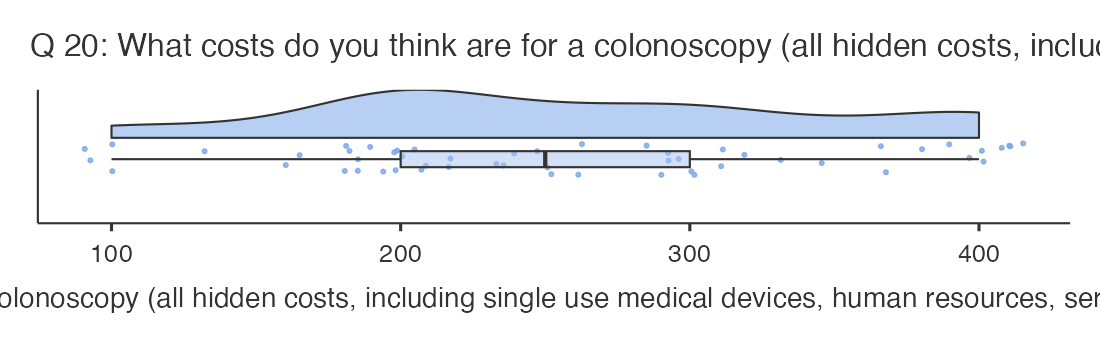


Figure C.1.36: Responses to question ” Q 20: What costs do you think are for a colonoscopy (all hidden costs, including single use medical devices, human resources, service and technologies depreciation, etc.)?”


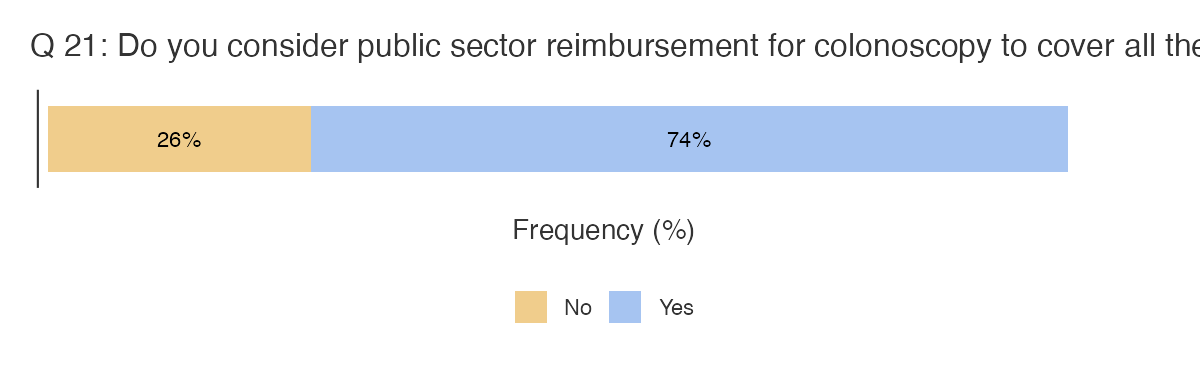


Figure C.1.37: Responses to question ” Q 21: Do you consider public sector reimbursement for colonoscopy to cover all the costs?”


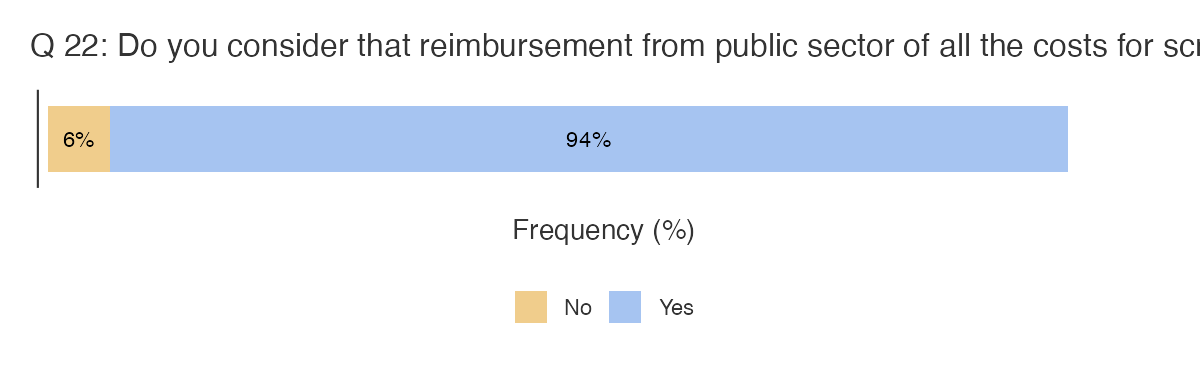


Figure C.1.38: Responses to question ”Q 22: Do you consider that reimbursement from public sector of all the costs for screening colonoscopy and early treatment will decrease the costs for healthcare system (decreasing the costs for medical care for patients with advanced disease, decreasing the burden of the disease in the society, increasing the contribution for society of treated patients, etc)?”


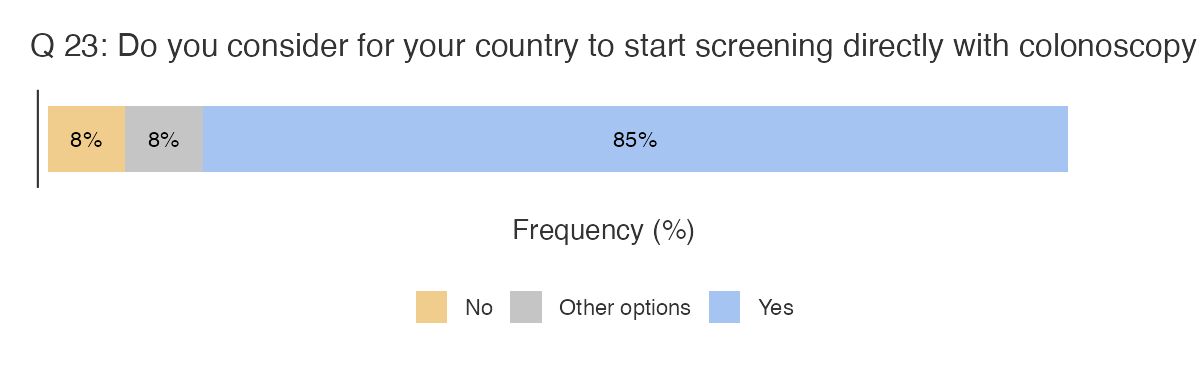


Figure C.1.39: Responses to question ” Q 23: Do you consider for your country to start screening directly with colonoscopy in high risk population, as defined by general practitioner?”


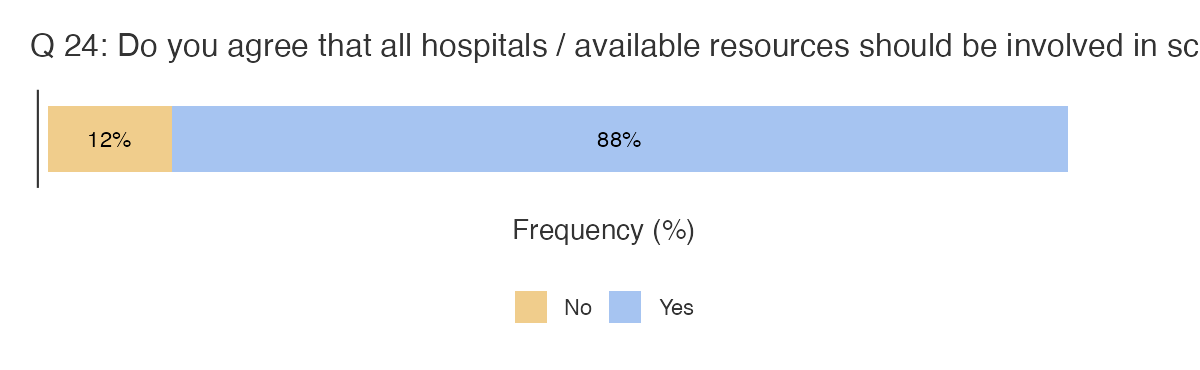


Figure C.1.40: Responses to question ” Q 24: Do you agree that all hospitals / available resources should be involved in screening?”


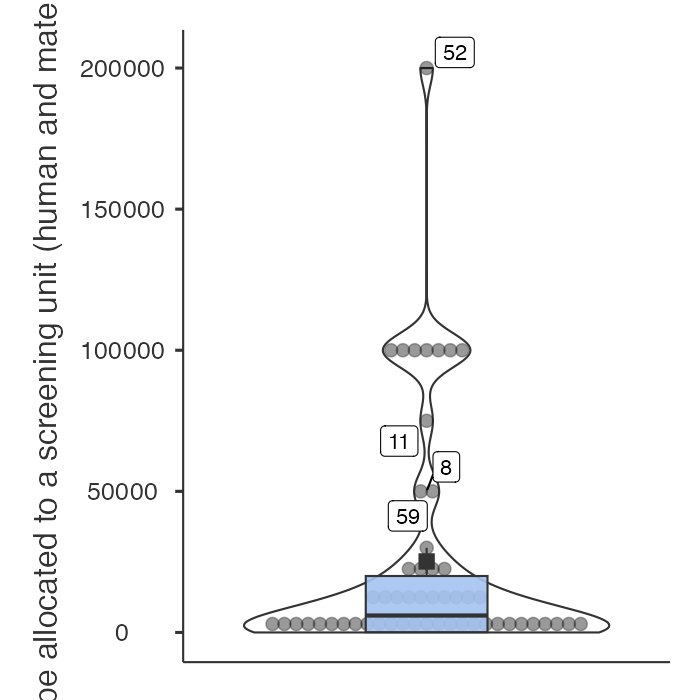


Figure C.1.41: Responses to question ” Q 25: What number of general populations should be allocated to a screening unit (human and material resources dedicated to screening in a hospital)?”

***Appendix C.2 - Figures of responses from the second voting round***


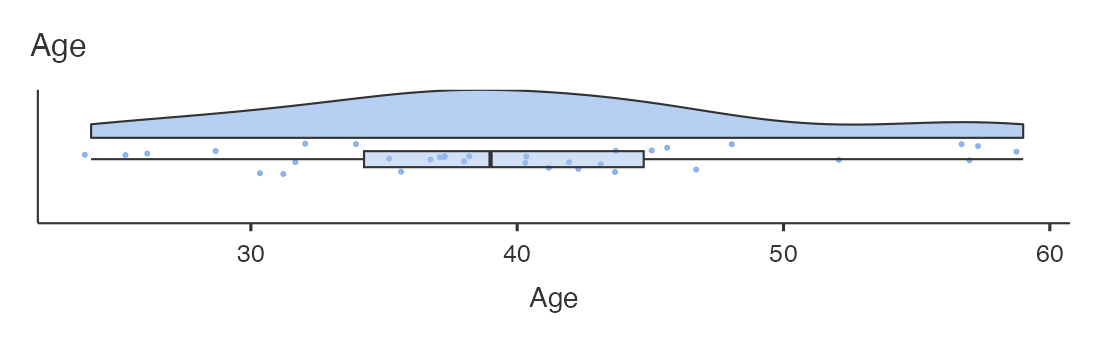


Figure C.2.1: Age of participants in the Delphi consensus.


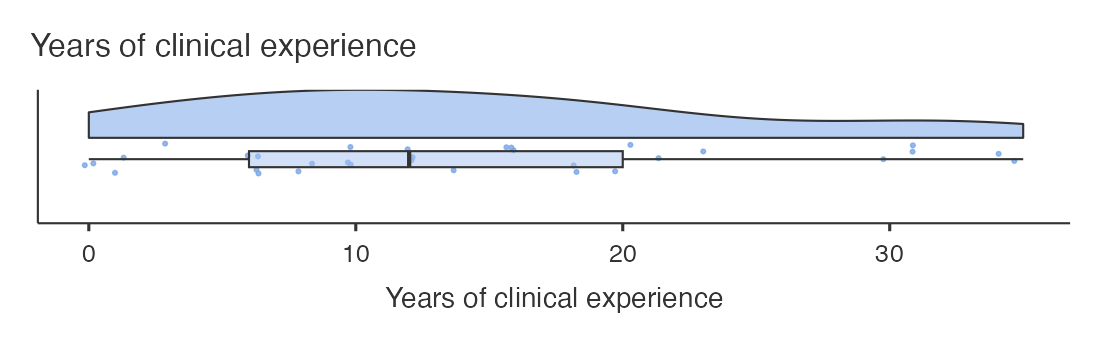


Figure C.2.2: Years of clinical experience of the participants in the Delphi consensus.


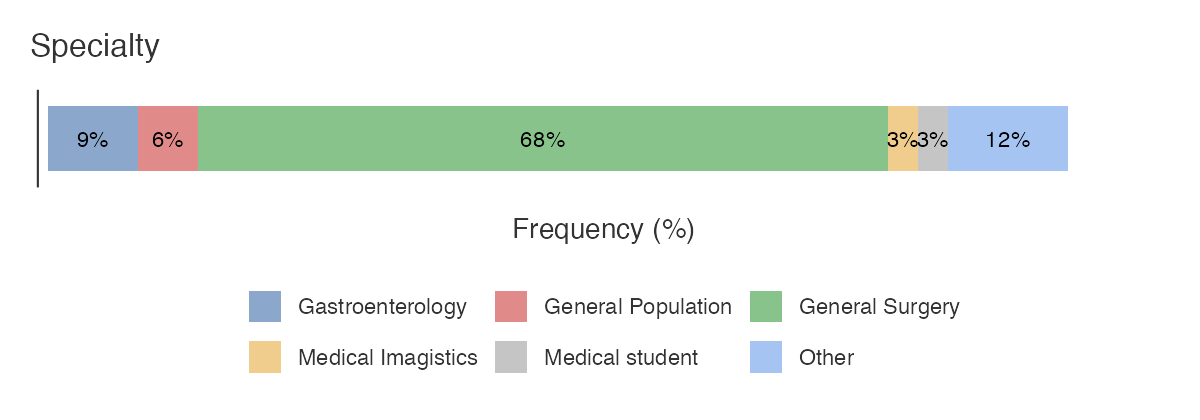


Figure C.2.3: Specialty of participants to the Delphi consensus


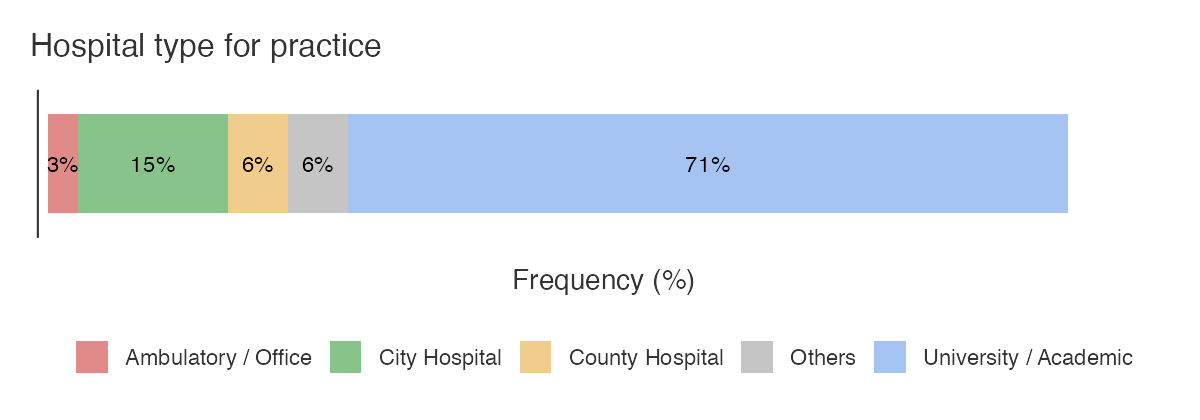


Figure C.2.4: Hospital type of participants to the Delphi consensus


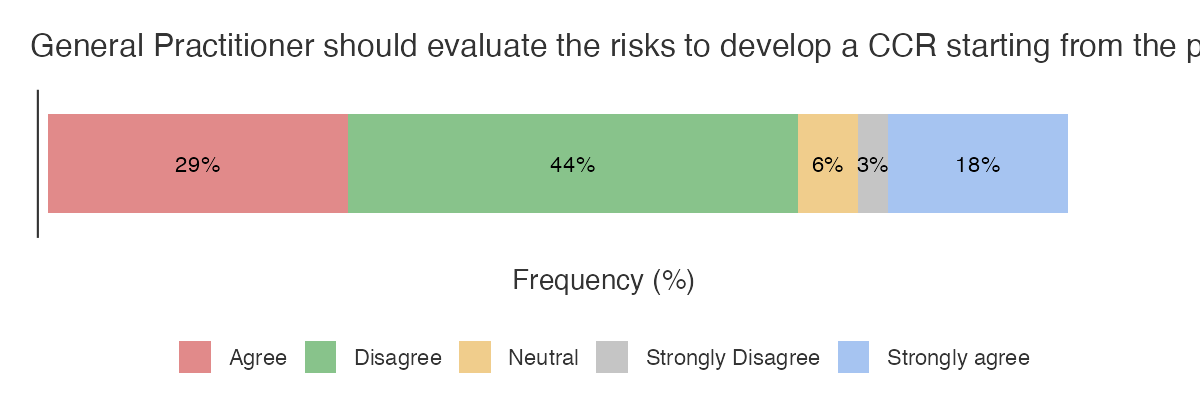


Figure C.2.5: Responses to question ” General Practitioner should evaluate the risks to develop a CCR starting from the patients age of 20-25 years old.”


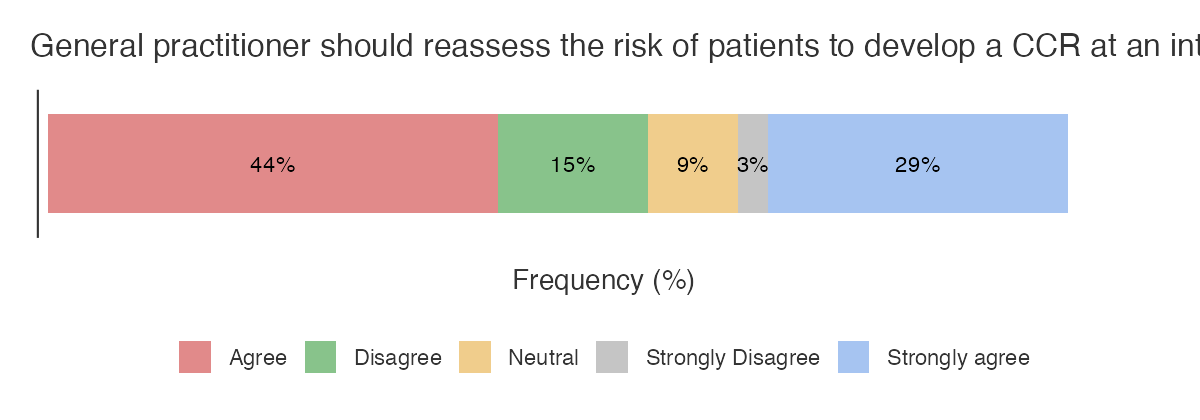


Figure C.2.6: Responses to question ” General practitioner should reassess the risk of patients to develop a CCR at an interval of 3 (three) years.”


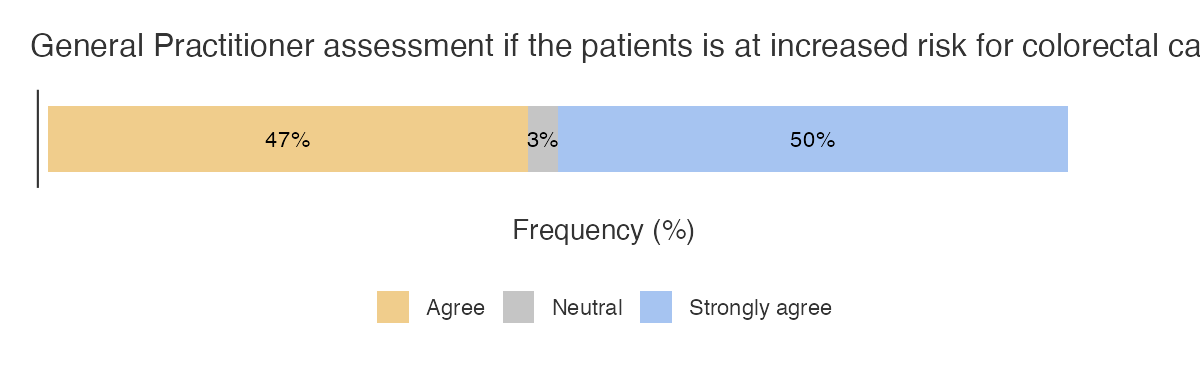


Figure C.2.7 Responses to question ” General Practitioner assessment if the patients is at increased risk for colorectal cancer should include as standard the following: (1) Familial history of cancer or adenomatous polyps; (2) Personal history of CRC or benign colorectal pathology such as adenomatous polyps; (3) Familial history of genetic syndromes associated with CRC; (4) Personal history of ulcerative colitis or Crohn disease; (5) Personal history of abdominal radiation for pediatric cancers.”


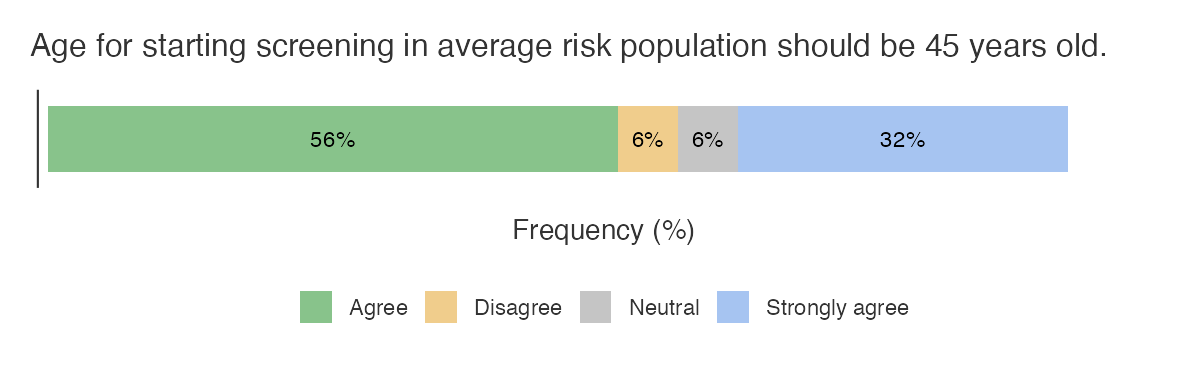


Figure C.2.8: Responses to question ” Age for starting screening in average risk population should be 45 years old.”

**References**

1. Clinical practice guidelines for the prevention, early detection and management of colorectal cancer - Cancer Council Australia. Preprint at https://files.magicapp.org/guideline/01c06572-c58e-4936-becb-ff45c842a7a0/published_guideline_9299-2_3.pdf (2017).

2. Goulding, M. Clinical practice guidelines for the prevention, early detection, and management of colorectal cancer: Population screening. *Cancer Council Australia* (2023).

3. Leddin, D. *et al.* Clinical practice guideline on screening for colorectal cancer in individuals with a family history of nonhereditary colorectal cancer or adenoma: The Canadian Association of Gastroenterology Banff consensus. *Gastroenterology* **155**, 1325-1347.e3 (2018).

4. Shaukat, A. *et al.* ACG clinical guidelines: Colorectal cancer screening 2021. *Am. J. Gastroenterol.* **116**, 458–479 (2021).

5. BCGuidelines.ca: Part 1: Screening for the Purposes of Colorectal Cancer Prevention and Detection in Asymptomatic Adults. Preprint at https://www2.gov.bc.ca/assets/gov/health/practitioner-pro/bc-guidelines/colorectalcancer-guideline-part1-final.pdf (2022).

6. Colorectal Cancer Screening (PDQ®). https://www.cancer.gov/types/colorectal/hp/colorectal-screening-pdq (2024).

7. Leddin, D. J. *et al.* Canadian Association of Gastroenterology position statement on screening individuals at average risk for developing colorectal cancer: 2010. *Can. J. Gastroenterol.* **24**, 705–714 (2010).

8. Wolf, A. M. D. *et al.* Colorectal cancer screening for average-risk adults: 2018 guideline update from the American Cancer Society. *CA Cancer J. Clin.* **68**, 250–281 (2018).

9. Colorectal cancer—clinician summary. https://canadiantaskforce.ca/colorectal-cancer-clinician-summary-2/.

10. Canadian Task Force on Preventive Health Care. Recommendations on screening for colorectal cancer in primary care. *CMAJ* **188**, 340–348 (2016).

11. Scottish Intercollegiate Guidelines Network: Diagnosis and management of colorectal cancer - A national clinical guideline. https://www.sign.ac.uk/media/1064/sign126.pdf (2016).

12. Qaseem, A. *et al.* Screening for Colorectal Cancer in Asymptomatic Average-Risk Adults: A guidance statement from the American College of Physicians (version 2). *Ann. Intern. Med.* **176**, 1092–1100 (2023).

13. Expert Panel on Gastrointestinal Imaging: *et al.* ACR appropriateness criteria® colorectal cancer screening. *J. Am. Coll. Radiol.* **15**, S56–S68 (2018).

14. Rex, D. K. *et al.* Colorectal Cancer screening: Recommendations for physicians and patients from the U.s. multi-Society Task Force on Colorectal Cancer. *Am. J. Gastroenterol.* **112**, 1016–1030 (2017).

15. Issaka, R. B., Chan, A. T. & Gupta, S. AGA clinical practice update on risk stratification for colorectal cancer screening and post-polypectomy surveillance: Expert Review. *Gastroenterology* **165**, 1280–1291 (2023).

16. Lopes, G. *et al.* Early detection for colorectal cancer: ASCO resource-stratified guideline. *J. Glob. Oncol.* **5**, 1–22 (2019).

17. Patel, S. G. *et al.* Updates on age to start and stop Colorectal Cancer screening: Recommendations from the U.s. multi-Society Task Force on Colorectal Cancer. *Gastroenterology* **162**, 285–299 (2022).

18. World Gastroenterology Organisation/International Digestive Cancer Alliance Practice Guidelines: Colorectal cancer screening. https://www.worldgastroenterology.org/UserFiles/file/guidelines/colorectal-cancer-screening-english-2007.pdf (2007).

19. US Preventive Services Task Force *et al.* Screening for colorectal cancer: US Preventive Services Task Force recommendation statement. *JAMA* **325**, 1965–1977 (2021).

20. Helsingen, L. M. *et al.* Colorectal cancer screening with faecal immunochemical testing, sigmoidoscopy or colonoscopy: a clinical practice guideline. *BMJ* **367**, l5515 (2019).
